# Characteristics of MDR *E*. *coli* strains isolated from Pet Dogs with clinic diarrhea: A pool of antibiotic resistance genes and virulence-associated genes

**DOI:** 10.1371/journal.pone.0298053

**Published:** 2024-02-28

**Authors:** Yu Yuan, Yan Hu, Xiaoli Zhang, Wenhao Zhong, Shulei Pan, Liqin Wang, Ziyao Zhou, Haifeng Liu, Shaqiu Zhang, Guangneng Peng, Ya Wang, Qigui Yan, Yan Luo, Keyun Shi, Zhijun Zhong

**Affiliations:** 1 College of Veterinary Medicine, Sichuan Agricultural University, Key Laboratory of Animal Disease and Human Health of Sichuan, Chengdu, China; 2 Jiangsu Yixing People’s Hospital, Yixing, China; 3 The Chengdu Zoo, Institute of Wild Animals, Chengdu, China; Bangladesh Agricultural University, BANGLADESH

## Abstract

The increasing number of multi-drug resistant (MDR) bacteria in companion animals poses a threat to both pet treatment and public health. To investigate the characteristics of MDR *Escherichia coli* (*E*. *coli*) from dogs, we detected the antimicrobial resistance (AMR) of 135 *E*. *coli* isolates from diarrheal pet dogs by disc diffusion method (K-B method), and screened antibiotic resistance genes (ARGs), virulence-associated genes (VAGs), and population structure (phylogenetic groups and MLST) by polymerase chain reaction (PCR) for 74 MDR strains, then further analyzed the association between AMRs and ARGs or VAGs. Our results showed that 135 isolates exhibited high resistance to AMP (71.11%, 96/135), TET (62.22%, 84/135), and SXT (59.26%, 80/135). Additionally, 54.81% (74/135) of the isolates were identified as MDR *E*. *coli*. In 74 MDR strains, a total of 12 ARGs in 6 categories and 14 VAGs in 4 categories were observed, of which *tetA* (95.95%, 71/74) and *fimC* (100%, 74/74) were the most prevalent. Further analysis of associations between ARGs and AMRs or VAGs in MDR strains revealed 23 significant positive associated pairs were observed between ARGs and AMRs, while only 5 associated pairs were observed between ARGs and VAGs (3 positive associated pairs and 2 negative associated pairs). Results of population structure analysis showed that B2 and D groups were the prevalent phylogroups (90.54%, 67/74), and 74 MDR strains belonged to 42 STs (6 clonal complexes and 23 singletons), of which ST10 was the dominant lineage. Our findings indicated that MDR *E*. *coli* from pet dogs carry a high diversity of ARGs and VAGs, and were mostly belong to B2/D groups and ST10. Measures should be taken to prevent the transmission of MDR *E*. *coli* between companion animals and humans, as the fecal shedding of MDR *E*. *coli* from pet dogs may pose a threat to humans.

## 1. Introduction

*Escherichia coli* (*E*. *coli*) is one of commensal microbiota in the gut of humans and animals [1]. With the widespread use of antimicrobials, the occurrence of multi-drug resistance (MDR) *E*. *coli* in humans and animals has posed a major threat to public health [2,3]. The presence of MDR *E*. *coli* in companion animals, such as pet dogs, undoubtedly raises concerns for both dogs and humans due to the high antimicrobial resistance (AMR) and the capability of carrying various antibiotic resistance genes (ARGs) of MDR strains [4,5]. Furthermore, previous studies have shown that there were associations between ARGs and virulence-associated genes (VAGs) existed in *E*. *coli*, and ARG-carrying strains may increase the likelihood of carrying VAGs [6]. However, only limited studies focused on MDR strains from pets in China, especially in Sichuan province which is considered as a major province for keeping pets in Southwest China [7].

In addition, recent studies have found that *E*. *coli* clones can be extensively shared between humans and household animals [8,9]. The transmission of high-risk *E*. *coli* clones between animals and humans has been recognized as a major public health issue [10,11]. For the population structure of *E*. *coli* clones, phylogenetic studies have shown that the B2 and D groups were more prevalent than the commensal groups (A and B1 groups) in *E*. *coli* isolates from diarrheic pet dogs [12,13]. Moreover, the multilocus sequence typing (MLST) was also used to analyze the population structure of *E*. *coli* clones [14]. Recent studies have identified a high population diversity of STs which related to zoonotic or pathotype in *E*. *coli* isolates from pet dogs, such as ST354, ST393, and ST457 *E*. *coli* were observed in companion animals from Australia [15]. And the high-risk ExPEC clones associated with humans and multidrug resistance, including sequence type (ST) 38, ST131, ST224, ST167, ST354, ST410, ST617 and ST648, have been identified in cats and dogs in Thailand which suggested there may be clonal dissemination between pets and humans [16].

In 2023, the number of domestic pets has exceeded 100 million in China [7]. Moreover, the latest annual report shows that the use of veterinary antibiotics for animals in China has exceeded 30,000 tons in 2020, tetracyclines, β-lactamases, and sulfonamides antimicrobial agents were widely used (Veterinary Bulletin of the Ministry of Agriculture and Rural People’s Republic of China, 2020) [17].With the widespread use of antimicrobials in animals, the high prevalence of MDR *E*. *coli* isolates from pet dogs has raised health concerns for both companion animals and humans [18,19]. To better understand the characteristics of MDR *E*. *coli* from pet dogs, we analyzed the antimicrobial resistance of *E*. *coli* isolates from diarrheal dogs, focusing on ARGs, VAGs, and population structure (ST and phylogenetic groups) for MDR *E*. *coli* to evaluate the potential threat.

## 2. Materials and methods

### 2.1 Sample collection

A total of 185 fresh feces samples were collected from pet dogs with clinical diarrhea during August 2021 to June 2022. Samples were excluded if the pet dogs were prescribed antimicrobial therapy or veterinary admission within the previous 3 months. The study was permitted by committee of Sichuan agricultural university (Permission number: DYY-2020303164) and the Sichuan Agricultural University Animal Ethical and Welfare Committee (Permission number: 20210268).

All samples were collected by professional veterinarians in Veterinary Teaching Hospital of Sichuan Agricultural University. Disinfection of hands and changing of disposable gloves were mandatory before sample collection. Samples were taken from the center of fresh feces by using sterile cotton swabs as soon as the pet dogs defecated to avoid cross-contamination from environmental bacteria on the ground. Then, samples were collected in sterile micro centrifuge tubes (Eppendorf) and were sent for processing to the laboratory within 12 h after collection.

### 2.2 Strains isolation

The isolation and identification of *E*. *coli* was performed as previous studies described [20,21]. Fecal samples were enriched in Luria-Bertani (LB) broth at 37°C, 120 r/min in a shaking incubator for 24 h. All isolates were identified presumptively by using phenotypic methods, including Gram staining, MacConkey agar growth and Eosin Methylene Blue agar growth. We further used 16S rDNA sequences (Primer: 5’-GAGTTTGATCCTGGCTCAG-3’; 5’-AGAAAGGAGGTGATCCAGCC-3’) [22] for final identification of *E*. *coli*. The confirmed isolates were stored in Luria-Bertani (LB) broth containing 50% glycerol at −20°C for further analysis.

### 2.3 Screening of MDR strains by antimicrobial susceptibility test

The antimicrobial susceptibilities of all isolates were tested using the standard disk diffusion method recommended by the Clinical and Laboratory Standards Institute (CLSI). A total of 16 antimicrobial agents in 6 categories as below were tested. aminoglycosides (gentamicin, CN, 10 μg; tobramycin, TOB, 10 μg), tetracyclines (tetracycline, TET, 30 μg; doxycycline, DOX, 30 μg), amide alcohols (chloramphenicol, C, 30 μg), quinolones (ciprofloxacin, CIP, 5 μg), sulfonamides (trimethoprim-sulfamethoxazole, SXT, 10 μg), β-lactams (ampicillin, AMP, 10 μg; cefazolin, KZ, 30 μg; cefuroxime, CXM, 30 μg; cefotaxime, CTX, 30 μg; cefepime, FEP, 30 μg; cefoxitin, FOX, 30 μg; aztreonam, ATM, 30 μg; imipenem, IPM, 10 μg; amoxicillin/clavulanic acid 2:1, AMC, 20 μg). For the 16 antimicrobial agents, the CN and TOB in aminoglycosides, DOX in tetracyclines, KZ, CTX, AMP, AMC in β-lactams and CIP in quinolones were used in pet animals at the location of our present study. The other antimicrobial agents (TET, C, CXM, FEP, ATM, IPM, FOX and SXT) have been found with *E*. *coli* resistance in dogs according to previous studies [23–25]. Results were interpreted in accordance with CLSI criteria (CLSI, 2023) [26]. *E*. *coli* ATCC25922 was used as a control. MDR strain was defined as being resistant to three or more antimicrobial categories [20].

### 2.4 DNA extraction and detection of ARGs, VAGs

Total genomic DNA of strains was extracted from isolates by using TIANamp Bacteria DNA kit (Tiangen Biotech, Beijing, China) following the manufacturer’s instructions. DNA samples were stored at -20°C for subsequent polymerase chain reaction (PCR) detection.

Primers of 23 ARGs in 6 categories (including 5 *bla*_CTX-M_ alleles for group1, 2, 8, 9, and 25) and 25 VAGs in 5 categories were synthesized by Huada Gene Technology Co. Ltd (Shenzhen, China). The PCR primer sequences and conditions for the ARGs and VAGs were showed in S1 and S2 Tables, respectively. PCR products were separated by gel electrophoresis in a 1.0% agarose gel in 1 × TAE buffer (40 mM Tris-acetate, 1 mM EDTA, pH 8.3), stained with GoldView^TM^ (Sangon Biotech, Shanghai, China), and photographed under ultraviolet light using the Bio-Rad ChemiDoc MP omnipotent imager (Bole, USA). All positive PCR products were sequenced with Sanger sequencing in both directions by Sangon Biotech (Shanghai, China). Sequences of ARGs and VAGs were analyzed online using the BLAST function of NCBI (http://blast.ncbi.nlm.nih.gov).

### 2.5 Phylogenetic grouping and MLST

Phylogenetic grouping (A/B1/B2/D) was determined for MDR *E*. *coli* isolates using PCR targeting *chuA*, *yjaA*, and *TSPE4*.*C2* following the protocol of Clermont et al. [27]. MLST was based on the sequencing results of seven housekeeping genes (*adk*, *fumC*, *gyrB*, *icd*, *mdh*, *purA* and *recA*) in each strain to obtain a numerical allelic profile which is abbreviated to a unique identifier of each strain: sequence type (ST) [28]. Allelic types of all seven housekeeping genes and ST of strains were determined following the protocol of the *E*. *coli* MLST database (https://pubmlst.org) [28,29]. The primer sequences of genes for MLST and phylogroups were shown in S3 Table. PCR products were separated by gel electrophoresis in a 1.0% agarose gel stained with GoldViewTM and photographed under ultraviolet light. All positive PCR products were sequenced with Sanger sequencing in both directions by Sangon Biotech (Shanghai, China). The sequences of housekeeping gene for MLST were analyzed online using pubMLST database (https://pubmlst.org).

The goeBURST algorithm in phyloviz 2.0 was used to clustering analysis of STs for MDR *E*. *coli* isolates, which divided the STs into several clusters, named as clonal complexes (CC), which consist of closely related STs with two allelic differences [30]. A clonal complex is typically composed of a single predominant genotype and closely relatives genotype [31].

### 2.6 Association analysis between ARGs and Antimicrobial resistance phenotypes (AMRs) or VAGs

The statistically analysis of data of AMR, ARGs and VAGs was conducted by using SPSS Statistics (version 26.0). *P*-value < 0.05 was considered to be statistically significant. Association analysis between ARGs and AMR or VAGs was performed by ggplot2 in RStudio (version 4.2.2; http://www.r-project.org) [32].

## 3. Results

### 3.1 AMRs of 135 isolates and Screening of MDR strains

**Fig 1:**
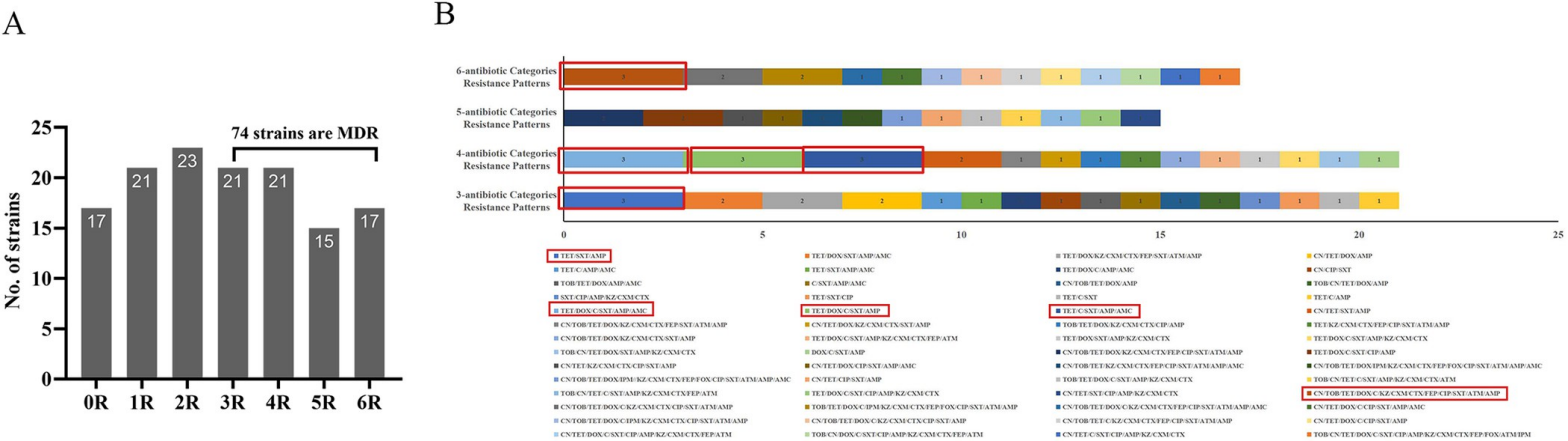
Antibiotic resistance patterns of *E*. *coli* isolates from pet dogs. (A) The abscissa 0R represents the strains that were sensitive to all antibiotics, and 1–6 R represents strains that were resistant to 1–6 antibiotic categories, respectively. Seventy-four *E*. *coli* isolates are MDR, of which 17 strains were resistant to 6 antibiotic categories; (B) Color bars demonstrate the distribution of phenotypic resistance patterns in 74 MDR *E*. *coli* isolates, and the Arabic number represent the number of strains. A total of 56 resistance patterns were observed by using disc diffusion assay. The red boxes highlight the prevalent resistant-phenotypes patterns (occurring three times), the other combinations (without red box) occurred only once or twice.

A total of 135 *E*. *coli* strains were successfully isolated from 185 fecal samples of diarrheal dogs. Among the 135 *E*. *coli* isolates, 118 strains (87.41%, 118/135) were resistant to at least one antimicrobial agent, while only 17 (12.59%, 17/135) strains were sensitive to all antimicrobial agents (Fig 1A). Among the 6 antibiotic categories, the resistance rate for β-lactam antibiotics had the highest resistance rats (76.30%, 103/135), followed by tetracyclines (64.44%, 87/135) and sulfonamides (59.26%, 80/135). The resistance rate to quinolones antibiotics was the lowest (25.93%, 35/135). Moreover, the resistance rates to AMP (71.11%, 96/135), TET (62.22%, 84/135), and SXT (59.62%, 80/135) were the top 3 in 16 antimicrobial agents. The lowest resistance rate was observed for FOX (3.70%, 5/135), and the resistance rates for remaining 12 antibiotics ranged from 4.44% (IPM) to 45.19% (DOX) (Table 1). We further analyzed the resistant-phenotype patterns of the 135 *E*. *coli* isolates, revealing that 54.81% (74/135) of isolates were identified as MDR *E*. *coli* (Fig 1A). Among the 74 MDR strains, 56 types of resistance phenotypic patterns were observed. The more common patterns were TET/SXT/AMP, TET/DOX/C/SXT/AMP/AMC, TET/DOX/C/SXT/AMP, TET/C/SXT/AMP/AMC, and CN/TOB/TET/DOX/C/KZ/CXM/CTX/FEP/CIP/SXT/ATM/AMP (Fig 1B).

**Table 1:** Antimicrobial resistance (AMR) detected in *E*. *coli* strains isolated from pet dogs (n = 135).

Category of antimicrobial	No. of Resistant Isolates (%)	Antibiotic		No. of Resistant Isolates (%)
β-lactams	103(76.30)	Penicillins	Ampicillin (AMP)	96 (71.11)
		1rd/2nd generation Cephalosporins	Cefazolin (KZ)	48 (35.56)
			Cefuroxime (CXM)	44 (32.59)
		3rd/4th generation Cephalosporins	Cefotaxime (CTX)	45 (33.33)
			Cefepime (FEP)	21 (15.56)
		β-lactam compound	Amoxicillin/clavulanic acid (AMC)	32 (23.70)
		Monobactams	Aztreonam (ATM)	25 (18.52)
		Carbapenems	Imipenem (IPM)	6 (4.44)
		Cephamicins	Cefoxitin (FOX)	5 (3.70)
Tetracyclines	87(64.44)	Tetracycline (TET)		84 (62.22)
		Doxycycline (DOX)		61 (45.19)
Sulfonamides	80(59.26)	Trimethoprim-sulfamethoxazole (SXT)		80 (59.26)
Aminoglycosides	45(33.33)	Gentamicin (CN)		39 (28.89)
		Tobramycin (TOB)		28 (20.74)
Amide alcohols	41(30.37)	Chloramphenicol (C)		41 (30.37)
Quinolones	35(25.93)	Ciprofloxacin (CIP)		35 (25.93)

### 3.2 Distribution of ARGs and VAGs in MDR *E*. *coli* strains

**Fig 2:**
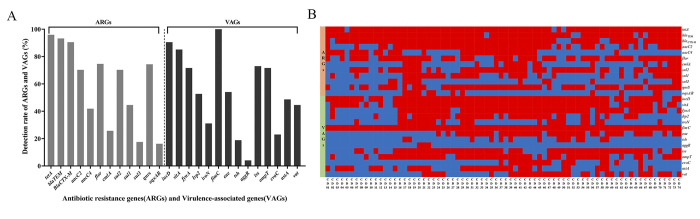
Distribution of antibiotic resistance genes (ARGs) and virulence-associated genes (VAGs) in 74 MDR *E*. *coli* strains from pet dogs. (A) The bar graphs show the detection rates of ARGs and VAGs. A total of 12 ARGs and 14 VAGs were detected, of which *tetA* (95.95%) and *fimC* (100%) were the most prevalent; (B) The abscissa represents the ID of MDR isolates and ordinate represents ARGs and VAGs. The red and blue regions represent the presence or absence of corresponding ordinate genes in an isolate, respectively. A high diversity of ARGs and VAGs was detected among MDR strains.

Twelve out of 18 ARGs in 6 categories were detected in our present study (Table 2 and Fig 2). The detection rate of *tetA* (95.95%, 71/74) was the highest, followed by *bla*_TEM_ (93.24%, 69/74) and *bla*_CTX-M_ (90.54%, 67/74). Moreover, detection rates of *flor*, *qnrS*, and *sul2* were all over 70% (74.32%, 74.32% and 70.27%, respectively). The detection rates of the remaining ARGs ranged from 16.22% (*oqxAB*) to 44.59% (*sul1*).

**Table 2:** Details of antimicrobial resistance (AMR) and antibiotic resistance genes (ARGs) detected in MDR *E*. *coli* strains isolated from pet dogs (n = 74).

Category of antimicrobial	No. of Resistant Isolates (%)	Antibiotics	No. of Resistant Isolates (%)	ARGs	No. of Positive Isolates (%)	Alleles of *bla*_TEM/_*bla*_CTX-M_ (%)
β-lactams	71 (95.95)	AMP	71 (95.95)	*bla*_TEM_	69(93.24)	*bla*_TEM-1_, 81.16 (56/69)
		KZ	38 (51.35)			*bla*_TEM-135_, 15.94 (11/69)
		CTX	38 (51.35)			*bla*_TEM-176_, 2.90 (2/69)
		CXM	38 (51.35)	*bla*_CTX-M_	67(90.54)	*bla*_CTX-M-14_, 79.10 (53/67)
		ATM	25 (33.78)			
		FEP	21 (28.38)			*bla*_CTX-M-65_, 4.45 (3/67)
		AMC	19 (25.67)			*bla*_CTX-M-55_, 31.34 (21/67)
		IPM	6 (8.11)			*bla*_CTX-M-64_, 7.46 (5/67)
		FOX	5 (6.76)			*bla*_CTX-M-15_, 5.97 (4/67)
Tetracyclines	71 (95.95)	TET	69 (93.24)	*tetA*	71(95.95)	--
		DOX	49 (66.22)			--
Sulfonamides	64 (90.54)	SXT	64 (90.54)	*sul2*	52(70.27)	--
				*sul1*	33(44.59)	--
				*sul3*	13(17.57)	--
Aminoglycosides	2 (56.76)	CN	37 (50.00)	*aacC2*	52(70.27)	--
		TOB	28 (37.84)	*aacC4*	31(41.89)	--
Amide alcohols	40 (54.05)	C	40 (54.05)	*flor*	55(74.32)	--
				*cmlA*	19(25.68)	--
Quinolones	34 (45.95)	CIP	34 (45.95)	*qnrS*	55(74.32)	--
				*oqxAB*	12(16.22)	--

We further analyzed the subtypes of *bla*_TEM_ and *bla*_CTX-M_, 3 variants of the *bla*_TEM_ gene and 5 variants of the *bla*_CTX-M_ gene were detected. Among the 3 variants of the *bla*_TEM_ gene, *bla*_TEM-1_ was the most frequent (81.16%, 56/69), followed by *bla*_TEM-135_ (15.94%, 11/69) and *bla*_TEM-176_ (2.90%, 2/69). For the *bla*_CTX-M_ gene, 3 variants in *bla*_CTX-M-1_ group (*bla*_CTX-M-55_, *bla*_CTX-M-15_ and *bla*_CTX-M-64_) and 2 variants in *bla*_CTX-M-9_ group (*bla*_CTX-M-65_ and *bla*_CTX-M-14_) were detected, *bla*_CTX-M-14_ (79.10%, 53/67) and *bla*_CTX-M-55_ (31.34%, 21/67) were more prevalent among the 5 variants observed, followed by *bla*_CTX-M-64_ (7.46%, 5/67), *bla*_CTX-M-15_ (5.97%, 4/67) and *bla*_CTX-M-65_ (4.45%, 3/67).

Fourteen out of 25 VAGs in 4 categories were detected in our study (Table 3 and Fig 2). The detection rate of *fimC* (100%, 74/74) was the highest, followed by *iucD* (90.54%, 67/74) and *sitA* (85.14%, 63/74). The detection rates of *iss* (72.97%, 54/74), *ompT* (71.62%, 53/74) and *fyuA* (71.62%, 53/74) were all above 70%. Detection rates of the remaining VAGs were ranged from 4.05% (*aggR*) to 54.05% (*eae*). Further analysis of the virulence determinants linked with different pathotypes revealed 3 DEC-related VAGs (*eae*, *aggR*, *astA*) and 11 ExPEC-related VAGs were detected. Notably, all MDR *E*. *coli* isolates carried at least one ExPEC-related VAG, 75.68% (56/74) of strains carried at least one DEC-related VAG, which also carried at least one ExPEC-related VAG. The average number of VAGs carried by per DEC-related strain (8.36) was significantly higher than that of DEC-unrelated VAGs strains (5.56) (*P* < 0.01).

**Table 3:** Detection rate of virulence-associated genes (VAGs) in MDR *E*. *coli* isolated from pet dogs (n = 74).

Category of virulence	VAGs	No. of Resistant Isolates (%)	Related pathotypes
Iron transport-related	*iucD*	67 (90.54)	ExPEC
	*sitA*	63 (85.14)	ExPEC
	*fyuA*	53 (71.62)	ExPEC
	*Irp2*	39 (52.70)	ExPEC
	*iroN*	23 (31.08)	ExPEC
Adhesion-related	*fimC*	74 (100.00)	ExPEC
	*eae*	40 (54.05)	DEC
	*tsh*	14 (18.91)	ExPEC
Invasion-and-toxin related	*vat*	33 (44.59)	ExPEC
	*astA*	36 (48.65)	DEC
	*aggR*	3 (4.05)	DEC
Antiserum survival factors-related	*iss*	54 (72.97)	ExPEC
	*ompT*	53 (71.62)	ExPEC
	*cvaC*	17 (22.97)	ExPEC

### 3.3 Associations between ARGs and AMRs or VAGs in MDR *E*. *coli* strains

Details of the detection rates of ARGs and AMR in 74 MDR *E*. *coli* strains were showed in Table 2. High prevalence of the β-lactam antibiotics genes (*bla*_TEM_ and *bla*_CTX-M_, 93.24% and 90.54%, respectively) were detected, and resistance rate of β-lactams antibiotic (AMP) was higher than 90% (71/74, 95.95%). For tetracyclines, *tetA* (71/74, 95.95%) was detected in MDR strains, and the proportions of strains with tetracyclines-resistant phenotypes were 95.95%. For sulfonamides, the resistant rate of SXT was 90.54% (64/74) in MDR strains, while detection rates of three related resistance genes (*sul1*, *sul2* and *sul3*) ranged from 17.57% (13/74) to 70.27% (52/74). For aminoglycosides, only two resistance genes (*aacC2* and *aacC4*) were detected with the detection rates ranging from 41.89% (31/74) to 70.27% (52/74), while the detection rate of MDR strains with aminoglycosides-resistant phenotype (CN and TOB) ranged from 37.87% (28/74) to 50% (37/74). Similarly, the detection rates of remaining antibiotic resistance phenotypes and related ARGs were fluctuated. Above results showed that only β-lactams and tetracyclines antibiotic-resistant phenotypes were generally matched with related ARGs in detection rates, but the others were not completely consistent.

**Fig 3:**
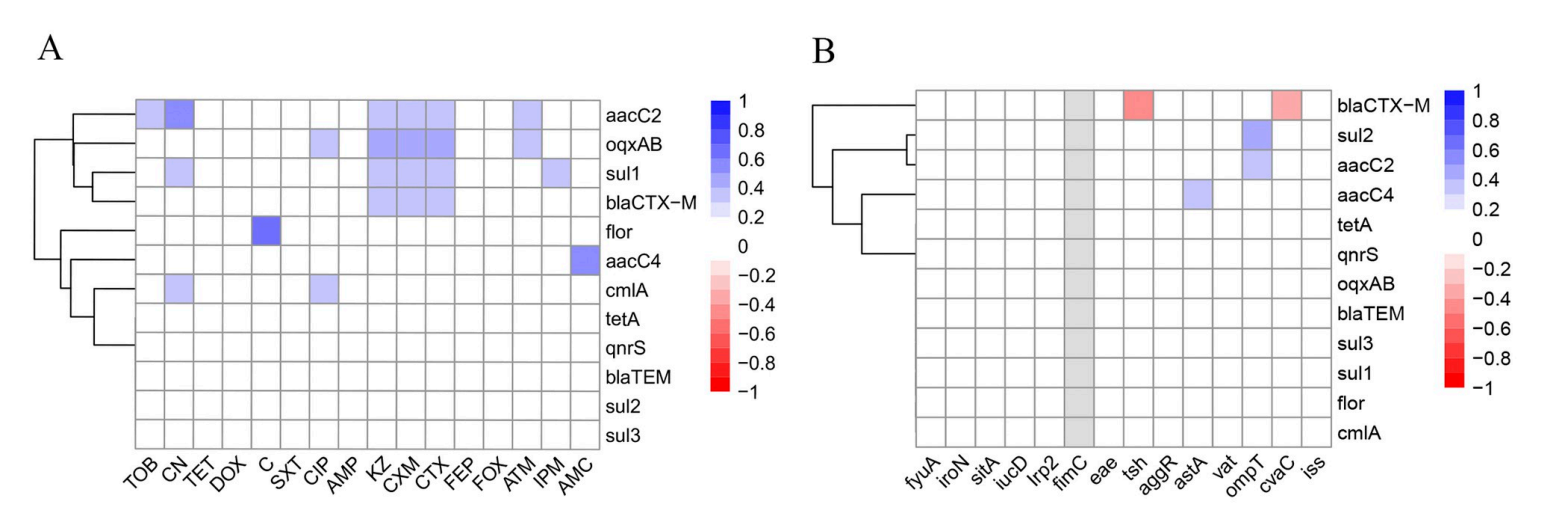
Heatmap of the correlation-coefficient (r) between ARGs and AMR or VAGs in 74 MDR *E*. *coli* strains from pet dogs. Blue indicates positive association (*r* > 0, *P* < 0.05) and red indicates negative association (*r* < 0, *P* < 0.05). The color scale on the right of figure indicates the *r*-valve: (A) Heatmap of the correlation coefficient between ARGs and AMRs. The color scale and corresponding *r*-valve indicate the association between corresponding abscissa AMRs and ordinate ARGs. Twenty-three positive association pairs were observed, of which the strongest association was found between *flor* and C; (B) Heatmap of correlation coefficient between ARGs and VAGs. The color scale and corresponding *r*-valve indicate the association between corresponding VAGs (abscissa) and ARGs (ordinate). Five association pairs were observed (3 association pairs were positive and 2 association pairs were negative), of which the strongest positive association was found between *ompT* and *sul2*, the strongest negative association was found between *bla*_CTX-M_ and *tsh*.

We further analyzed the associations between ARGs and AMR in 74 MDR strains. As shown in Fig 3A, a total of 23 positive association pairs (*r* > 0, *P* < 0.05) were observed between ARGs and AMR, of which the association between amide alcohols antibiotic resistance gene *flor* and amide alcohols-resistant phenotype (C) was the strongest. Moreover, the associations between 12 ARGs and 14 VAGs were further analyzed and the results were showed in Fig 3B, only 5 association pairs were observed, of which 3 pairs (*sul2/ompT*, *aacC2/ompT*, *aacC4/astA*) showed positive association (*r* > 0, *P* < 0.05), and the strongest association pair was observed between *sul2* and *ompT*. The other 2 pairs (*bla*_CTX-M_/*tsh*, *bla*_CTX-M_/*cvaC*) were negative associations (*r* < 0, *P* < 0.05), and the strongest negative association pair was observed between *bla*_CTX-M_ and *tsh*.

### 3.4 Phylogenetic Grouping and MLST of 74 MDR *E*. *coli* isolates

**Fig 4:**
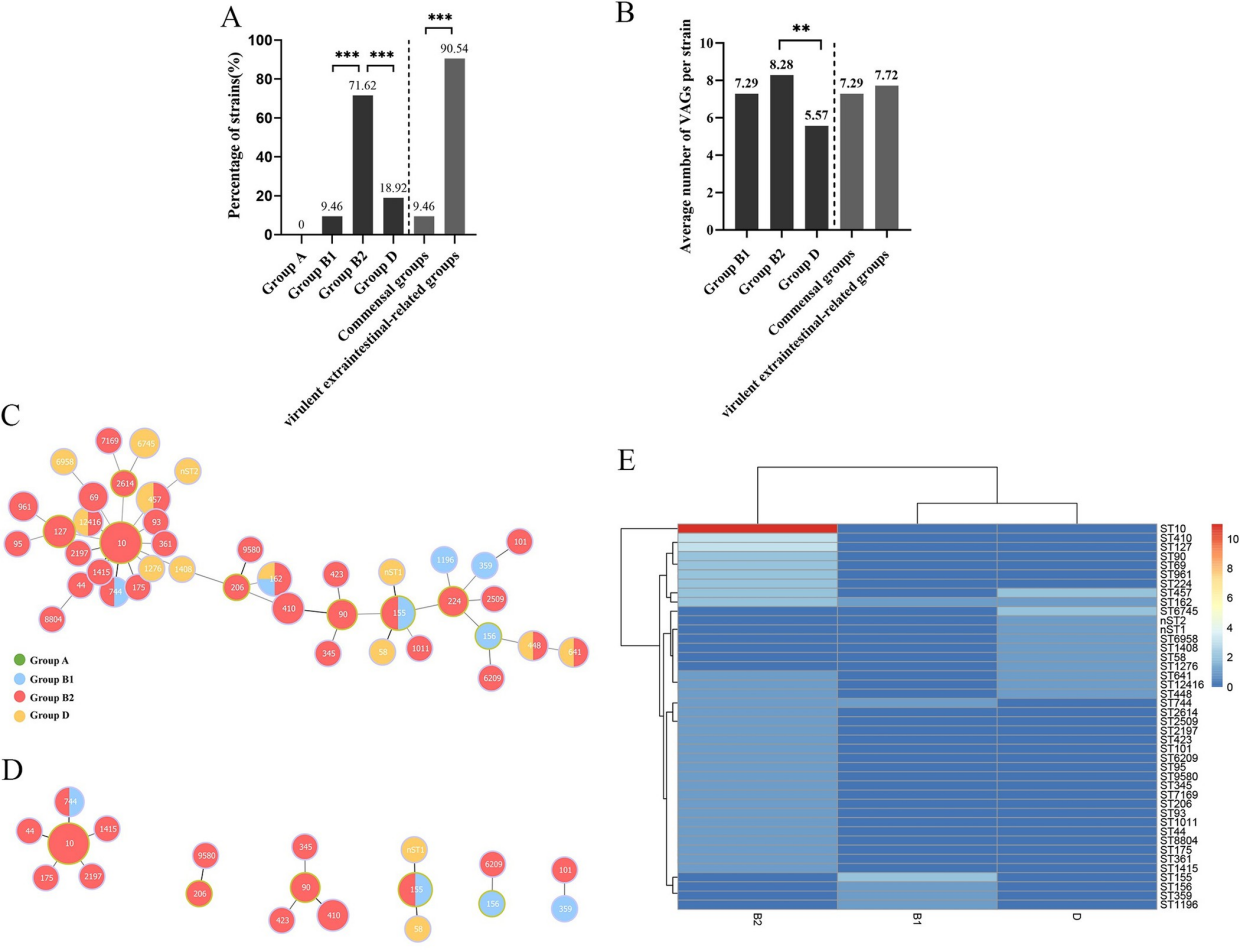
Distribution of phylogenetic groups and STs in 74 MDR *E*. *coli* strains from pet dogs. (A) The distribution of phylogroups in MDR strains. Commensal groups included groups A and B1, and virulent extraintestinal-related groups included groups B2 and D. Marking * represents a significant difference, *, *P* < 0.05; **, *P* < 0.01; ***, *P* < 0.001. Significantly, the virulent groups were the most prevalent; (B) The average number of VAGs per isolate in each phylogroup. Marking * represents significant difference, *, *P* < 0.05; **, *P* < 0.01; ***, *P* < 0.001. Obviously, the highest value observed was 8.28 in group B2.; (C) Minimum spanning tree of MLST types in MDR strains. The circle size indicates the proportion of isolates belonging to the ST. The color within each circle represents phylogroups and indicates the proportion of isolates belonging to different phylogroups. Each link between circles indicates one mutational event and the distance is scaled as the number of allele differences between STs. The yellow-green outlines of the circles represent the ST is the founder ST of one clonal complex (CC), and the other STs (with purple outlines of the circles) of the CC are derived from the founder ST with two allelic differences. The 74 MDR strains exhibited a high diversity of STs (42 STs were identified) in our present study and ST10 was the most prevalent; (D) The clonal complexes (CCs) among the 42 STs. Forty-two STs were clustered into 6 clonal complexes, and the remaining 23 STs were single. ST-10CC was the most prevalent lineage containing 6 STs; (E) Heatmap demonstrates the distribution of STs and phylogroups. The color scale and corresponding value indicate the number of *E*. *coli* isolates belonging to the corresponding phylogroups (abscissa) and STs (ordinate). Blue indicates a low number of strains, white indicates an intermediate value, and red indicates a high number of strains. As the heatmap shows, B2-ST10 was the most prevalent clone.

As shown in the Fig 4A, group B2 (71.62%, 53/74) was the most prevalent in 74 MDR *E*. *coli* strains, followed by group D (18.92%, 14/74) and group B1 (9.46%, 7/74). 90.54% (67/74) of the MDR *E*. *coli* strains belonged to virulent extraintestinal-related group (B2 and D) and only 9.46% strains belonged to commensal group (B1) (*P* < 0.001). Furthermore, we analyzed the average number of VAGs carried by per strain in different groups, of which the highest observed was 8.28 in group B2, followed by 7.29 in group B1 and 5.57 in group D (*P* < 0.01). And no significant difference (*P* > 0.05) was observed in the average number of VAGs carried by per strain between commensal group (B1) and virulent extraintestinal-related groups (B2/D) (Fig 4B).

In addition, 42 different sequence types (STs) were identified in 74 MDR *E*. *coli* isolates (Fig 4C). Eleven strains belonged to ST10, followed by ST155 (4 strains), ST162 (4 strains), ST457 (4 strains), ST127 (3 strains) and ST410 (3 strains), the remaining 36 STs contained only one strains, respectively. Moreover, two new sequence types (nST1, nST2) were identified in our present study (S4 Table). By using goeBURST algorithm, 42 STs were clustered into 6 clonal complexes (CCs) and 23 singletons. Among the 6 clonal complexes, ST44, ST175, ST744, ST1415 and ST2197 were included in clonal complex 10CC with the founder ST10 (ST-10CC), and ST-10CC was the predominant lineage containing 17 MDR strains. ST345, ST410 and ST423 belonged to 90CC with the founder ST90 (ST-90CC); ST58, ST155 and nST1 belonged to 155CC with founder ST155 (ST-155CC); ST6209 and ST156 belonged to one clonal complex with the founder ST156 (ST-156CC), ST9580 and ST206 belonged to one clonal complex with the founder ST206 (ST-206CC), respectively. However, the CC contained ST359 and ST101 without any founder ST (Fig 4D). The heatmap of phylogroups and MLST distribution showed that 11 (14.86%, 11/74) strains simultaneously belonged to B2 group and ST10, which were the most prevalent clones in our present study (Fig 4E).

## 4. Discussion

The widespread use of antibiotics has significantly caused the increase of MDR *E*. *coli* which were isolated from companion animals [33]. To understand the characteristics of MDR strains and evaluate the potential threat, we detected the AMR of 135 *E*. *coli* isolates from pet dogs and further screened ARGs, VAGs, phylogenetic grouping and MLST for 74 MDR strains.

One hundred and thirty-five *E*. *coli* isolates from pet dogs showed high resistance to TET, AMP and SXT, which was consistent with another study in China [34]. Notably, TET has been used for treating animal infection disease and has also been used as “growth promoters” or “feed efficiency products” for a long time [35,36], which may activate the resistance of TET in *E*. *coli*. Once the antimicrobial-resistant *E*. *coli* occurs, it exists for a long time due to hard elimination [37]. In addition, TET resistance has been widely observed in *E*. *coli* from animals, such as dogs [13,24], yaks [38] and pigs [39], and the antibiotic resistance genes encoding AMR can be transmitted through environment and mobile genetic elements [40–42], which may be one of the reasons for high TET-resistant *E*. *coli* detected in our present study, even though TET has not been used in the area recently. The similar phenomenon was also found in the resistant strains to 3rd/4th generation cephalosporins, fluoroquinolones and carbapenems antibiotics in our present study, which have been advocated to avoid or restrict use in veterinary antimicrobial selection in China [34,43]. The resistance rates of AMP and SXT were consistent with the results from pet dogs in Nigeria, Australia and Brazil [19,23,44]. According to previous studies, frequent use of antimicrobial agents in clinics may be responsible for the high resistance to AMP and SXT [45,46], the β-lactam antibiotics have also been widely used in the location of the present study, and SXT is one of the most animal-used antimicrobials in China [45]. In our study, we further screened 74 MDR strains from 135 *E*. *coli* isolates. Compared with previous studies in Sichuan, the detection rate of MDR strains among *E*. *coli* isolates from pet dogs decreased from 100% in 2017 [47] and 70% in 2018 [48] to 54.81% in our study during 2021–2022, which was similar to the trend observed in Northeast China (which decreased from 76.92% in 2012–2013 to 62.42% in 2021) [43]. The decrease in the MDR detection rate may be due to that the relevant authorities of China have released several policies to curb the increase in antimicrobial resistance in animal-derived bacteria [43]. Although the detection rate of MDR *E*. *coli* from pet dogs in Sichuan has decreased, it is still higher than that detected in other countries [44,49]. Overall, resistant strains may have been circulating in the dog population long before the antibiotic usage restriction were introduced, and AMR strains are currently difficult to eliminate. The high detection rates of MDR *E*. *coli* in our study implied that more effective measures should be taken to control the occurrence and spread of MDR *E*. *coli* from companion animals.

It is well-known that the antibiotic resistant phenotype of bacteria is related to ARGs [50]. Therefore, we further analyzed the distribution of ARGs in 74 MDR strains. In our study, 12 out of 18 ARGs were detected in 74 MDR strains (detection rates ranged from 16.22% to 95.95%), of which *tetA*, *bla*_TEM_ and *bla*_CTX-M_ were the dominant ARGs. Notably, the detection rate of *tetA* in our study was higher than previous studies from dogs in China which ranged from 28% to 85.08% [43,51,52]. The *bla*_TEM_ and *bla*_CTX-M_ were the prevalent β-lactam resistance genes in our study which were consistent with previous study for *E*. *coli* from dogs in China [43]. We further analyzed the prevalent subtypes of *bla*_CTX-M_ genes, *bla*_CTX-M-55_ (in *bla*_CTX-M-1_ group) and *bla*_CTX-M-14_ (in *bla*_CTX-M-9_ group) were more prevalent. Among variants of *bla*_CTX-M-9_ group, *bla*_CTX-M-14_ has been considered as a common CTX-M variant worldwide, especially in China, Korea and Japan [53]. A high prevalence of *bla*_CTX-M-14_ was also observed in our present study (79.10%). Notably, *bla*_CTX-M-14_ was usually found on plasmid (such as IncF and IncK) according to previous studies [54,55]. The high prevalence of *bla*_CTX-M-14_-positive strains observed in our present study indicated a high risk of potential transmission. For variants of the *bla*_CTX-M-1_ group, *bla*_CTX-M-55_ has become the second most common CTX-M subtype in Chinese clinical *E*. *coli* isolates after *bla*_CTX-M-14_ [54], which was also dominant in our present study. Furthermore, Cottell et al. has observed the frequent clonal transmission of *bla*_CTX-M-55_-positive *E*. *coli* between different hosts (humans and animals such as duck, chicken, and swine) [54]. The high prevalence of *bla*_CTX-M-14_-positive and *bla*_CTX-M-55_-positive strains observed in our present study suggests more studies should focus on the capability of clonal transmission between different hosts in the future.

Increasing studies proposed that the relationship between AMRs and ARGs is not completely consistent [22,56]. The detection rates of β-lactam and tetracycline antibiotics resistant phenotypes were generally consistent with the related ARGs. However, for sulfonamides, aminoglycosides, amide alcohols, and quinolones, the detection rates of related ARGs were fluctuated, e.g., the resistant rate of sulfonamides was 90.54% in MDR strains, while the detection rates of *sul* ranged from 17.57% to 70.27%. Moreover, we further analyzed the statistical associations between ARGs and AMRs, 23 positive association pairs were observed in MDR strains. Only *flor* and C, *bla*_CTX-M_ and CTX/CXM/KZ, *oqxAB* and CIP, *aacC2* and TOB/CN showed consistency in ARG and related AMR. The other 16 association pairs were not completely consistent (Fig 3A). The similar phenomenon was also found in *E*. *coli* from captive non-human primates [22] and waterfowl [56] in China which indicated the different expression of ARGs, such as the abnormal expression of ARGs and the expression of ARGs has not reached that level which can activate the antibiotic resistance, may be a possible reason the incomplete consistency between AMR and ARGs [56].

Recent studies have shown that there was an association between ARGs and VAGs, and have proposed that they were linked and interacted with each other [6,56]. In order to explore the virulence traits and the association of ARGs and VAGs in MDR *E*. *coli*, we analyzed the distribution of VAGs in MDR strains. In our present study, 25 VAGs were selected for detection according to previous studies, among which 12 VAGs (*eae*, *papA*, *bfpA*, *aggR*, *pic*, *astA*, *ipaH*, *stx1*, *stx2*, *elt*, *esta and estb*) are related to Diarrhoeagenic *Escherichia coli* (DEC) [57–59], the remaining 13 VAGs (*fyuA*, *iroN*, *sitA*, *iucD*, *Irp2*, *fimC*, *tsh*, *vat*, *ompT*, *cvaC*, *iss*, *hlyF*, *hlyA*) are related to Extraintestinal *Escherichia coli* (ExPEC) [60–62]. Eleven ExPEC-related VAGs and 3 DEC-related VAGs (*eae*, *astA*, *aggR*) were detected in our present study. And the detection rates of VAGs in our study, which ranged from 4.99% to 100%, were higher than previous studies in dogs from Shaanxi and Shandong, China (ranging from 0.6% to 81.6% in Shaanxi and 2.53% to 87.34% in Shandong, respectively) [34,63]. Moreover, 75.68% (56/74) of the MDR strains simultaneously combined at least one ExPEC-related and DEC-related VAG, indicating the MDR strains in our present study harbored VAGs related to different pathotypes and could be considered as potentially virulent hybrid pathogenic strains. MDR strains with potential hybrid pathogen detected in pet dogs will pose a threat to other companion animals and their owners.

In addition, the association between VAGs and ARGs among MDR strains was further analyzed. Five associated pairs were observed, of which 3 pairs were positive and 2 pairs were negative. Compared with other studies of *E*. *coli* from giant pandas (46 associated pairs, of which 45 pairs were positive and 1 pair was negative) [6] and waterfowl (43 associated pairs, of which 36 pairs were positive and 7 pairs were negative) [56], the associations observed between ARGs and VAGs in MDR strains from pet dogs were not common in our present study. The possible reasons for drawing associations between VAGs and ARGs may be related to the co-location on the same mobile genetic elements, while negative associations indicated gene incompatibilities [64]. Recent studies also showed that many ARGs were inserted into conjugative plasmids carrying VAGs, which may provide conditions for drawing positive associations between ARGs and VAGs [65]. In our study, the strongest positive association was observed between *ompT* and *sul2*, which is usually detected in plasmids, indicating the positive association between *ompT* and *sul2* may be related to the plasmid [40,66]. Similarly, the location of *aacC*, which usually was detected in the variable region of integron, may be related to the other two positive associations (*aacC2*/*ompT*, *aacC4*/*astA*) observed in our present study [5]. The positive association between ARGs and VAGs implied the high risk of plasmid-mediated co-transmission of ARGs and VAGs in *E*. *coli*, which could accelerate the spread of VAGs and ARGs within *E*. *coli* populations and enhance the emergence possibility of new pathogens with increased virulence and resistance potential. Negative associations observed in our present study may also be related to mobile genetic elements. For example, the VAG *cvaC* is usually found in virulent IncF plasmids [66], while *bla*_CTX-M_ is usually found in integron [67], the different locations on mobile genetic elements may be related to the negative associations between *bla*_CTX-M_ and *cvaC* in our present study. While the specific mechanism for the associations observed in our study required more comprehensive studies to validate.

Previous studies have shown that groups A and B1 are considered to be commensal groups, and groups B2 and D are considered to be virulent extraintestinal-related groups [27], which have been classified as potentially pathogenic [5,6]. A study from Iran showed that resistant *E*. *coli* strains mostly belonged to groups B2 and D [68]. Similarly, our present results showed a high prevalence of strains belonging to virulent extraintestinal-related groups (B2 and D) were detected among MDR *E*. *coli* from pet dogs. According to previous study, phylogenetic grouping is closely related to virulence genes (group A and B1 strains carry fewer virulence genes, while group B2 and D strains carry more virulence genes) [6], whereas in our present study, no significant difference was observed in the average number of VAGs carried by per strains between commensal groups and virulent extraintestinal-related groups. These phenomena suggest that antimicrobial resistance may increase the possibility of carrying more virulent factors (for example, the number of VAGs carried) in *E*. *coli* strains [69], implying that highly antibiotics resistant intestinal *E*. *coli* strains may also lead to extraintestinal infections which could be an emergent public health issue for humans, animals and the environment [70,71].

At last, we used MLST to analyze the dominant lineage of MDR strains, and our results showed that 74 MDR strains exhibited a high population diversity of STs (42 STs were identified), of which ST10 was the most prevalent. Another study of *E*. *coli* from dogs in China has found that the pandemic clone was ST131 (accounting for 9.8%) [63], which was identified as the most common ST in ExPEC, covering all geographical regions [72]. Moreover, studies from Australia and Thailand showed that ST354 and ST410 were the dominant clones in *E*. *coli* isolates from dogs, respectively [15,16]. In our study, the most common ST was ST10 (occurring in 14.86% of strains) which was different from the above studies, indicating that the prevalent ST lineage of *E*. *coli* isolates from pet dogs may vary in different geographical regions. ST10 was widely detected in *E*. *coli* from other animals in China, such as pigs [73] and chickens [74], indicating that ST10 may be the prevalent ST in *E*. *coli* strains from animals in China. Moreover, ST10 strains mostly belonged to B2 groups in our present study, and this phenomenon was also observed in *E*. *coli* isolates from chickens [74] and in the ExPEC isolates from humans [75]. In other studies, ST10 strains identified from pigs [73] and humans [76] mostly belonged to group A or D. This phenomenon indicated that the relationship between ST and phylogroups may vary in *E*. *coli* isolated from different hosts.

## 5. Conclusions and limitations

Seventy-four MDR strains were observed among 135 *E*. *coli* isolates from pet dogs, of which 12 ARGs and 14 VAGs were detected, implying that the *E*. *coli* isolates can be considered as a pool of ARGs and VAGs. Moreover, groups B2 and D, which are classified as potentially pathogenic, were predominant in the MDR isolates, and ST10 was the prevalent clonal lineage. Our findings have important implications for a preliminary understanding of the characteristics of MDR *E*. *coli* from diarrheal dogs and evaluating the potential risk of resistance and virulence in canine *E*. *coli*.

In our present study, the number of samples included was limited and the sampling site was only at our Veterinary Teaching Hospital. Future investigations should include more samples and expand sampling areas. Moreover, the use of High-throughput Sequencing or Whole Genome Sequencing could provide more comprehensive information on MDR *E*. *coli* strains from diarrheal dogs.

## Supporting information

S1 Table PCR primer and conditions for antibiotic resistance genes (ARGs) used in this study. (XLSX)

S2 Table PCR primer and conditions for virulence-associated genes (VAGs) used in this study. (XLSX)

S3 Table PCR primer and conditions of 7 housekeeping genes for MLST and 3 related genes for phylogroups used in this study. (XLSX)

S4 Table Distribution of sequence types (STs) in MDR *E*. *coli* strains from pet dogs (n = 74). (XLSX)

S5 Table Details of STs, phylogroups, AMR, ARGs and VAGs pattern for MDR *E*. *coli* isolated from pet dogs. (XLSX)

S6 Table The performance Standards for Antimicrobial Susceptibility testing in the present study. (XLSX)

S7 Table Clinical history of the pet dogs which successfully isolated *E*. *coli* strains in our present study. (XLSX)

## References

[1] Foster-Nyarko E, Pallen MJ. The microbial ecology of *Escherichia coli* in the vertebrate gut. FEMS Microbiology Reviews. 2022 May 6;46(3):fuac008. 35134909 10.1093/femsre/fuac008 PMC9075585

[2] Ferri M, Ranucci E, Romagnoli P, Giaccone V. Antimicrobial resistance: A global emerging threat to public health systems. Critical Reviews in Food Science and Nutrition. 2017 Sep 2;57(13):2857–76. doi: 10.1080/10408398.2015.1077192 26464037

[3] Pomba C, Rantala M, Greko C, Baptiste KE, Catry B, Van Duijkeren E, et al. Public health risk of antimicrobial resistance transfer from companion animals. J Antimicrob Chemother. 2016 Dec 20;dkw481. 10.1093/jac/dkw481 27999066

[4] Kennedy CA, Walsh C, Karczmarczyk M, O’Brien S, Akasheh N, Quirke M, et al. Multi-drug resistant Escherichia coli in diarrhoeagenic foals: Pulsotyping, phylotyping, serotyping, antibiotic resistance and virulence profiling. Veterinary Microbiology. 2018 Sep;223:144–52. doi: 10.1016/j.vetmic.2018.08.009 30173740

[5] Karczmarczyk M, Abbott Y, Walsh C, Leonard N, Fanning S. Characterization of Multidrug-Resistant Escherichia coli Isolates from Animals Presenting at a University Veterinary Hospital. Appl Environ Microbiol. 2011 Oct 15;77(20):7104–12. doi: 10.1128/AEM.00599-11 21856835 PMC3194860

[6] Fan S, Jiang S, Luo L, Zhou Z, Wang L, Huang X, et al. Antibiotic-Resistant Escherichia coli Strains Isolated from Captive Giant Pandas: A Reservoir of Antibiotic Resistance Genes and Virulence-Associated Genes. Veterinary Sciences. 2022 Dec 18;9(12):705. doi: 10.3390/vetsci9120705 36548866 PMC9786197

[7] Wang B, Li S, Liang G, Li J, Li Y. Current status and prospects of pet medical development in Sichuan province. Anim. Agriculture. 2023 418; (01): 59–62.

[8] Johnson JR, Clabots C. Sharing of Virulent Escherichia coli Clones among Household Members of a Woman with Acute Cystitis. Clinical Infectious Diseases. 2006 Nov 15;43(10):e101–8. doi: 10.1086/508541 17051483

[9] Naziri Z, Poormaleknia M, Ghaedi Oliyaei A. Risk of sharing resistant bacteria and/or resistance elements between dogs and their owners. BMC Vet Res. 2022 Dec;18(1):203. doi: 10.1186/s12917-022-03298-1 35624502 PMC9137046

[10] Zhai W, Wang T, Yang D, Zhang Q, Liang X, Liu Z, et al. Clonal relationship of *tet* (X4)-positive *Escherichia coli* ST761 isolates between animals and humans. Journal of Antimicrobial Chemotherapy. 2022 Jul 28;77(8):2153–7. 35678277 10.1093/jac/dkac175

[11] Nandanwar N, Janssen T, Kühl M, Ahmed N, Ewers C, Wieler LH. Extraintestinal pathogenic Escherichia coli (ExPEC) of human and avian origin belonging to sequence type complex 95 (STC95) portray indistinguishable virulence features. International Journal of Medical Microbiology. 2014 Oct;304(7):835–42. doi: 10.1016/j.ijmm.2014.06.009 25037925

[12] Vega-Manriquez XD, Ubiarco-López A, Verdugo-Rodríguez A, Hernández-Chiñas U, Navarro-Ocaña A, Ahumada-Cota RE, et al. Pet dogs potential transmitters of pathogenic Escherichia coli with resistance to antimicrobials. Arch Microbiol. 2020 Jul;202(5):1173–9. doi: 10.1007/s00203-020-01828-9 32076735

[13] Karahutová L, Mandelík R, Bujňáková D. Antibiotic Resistant and Biofilm-Associated Escherichia coli Isolates from Diarrheic and Healthy Dogs. Microorganisms. 2021 Jun 19;9(6):1334. doi: 10.3390/microorganisms9061334 34205399 PMC8234098

[14] Riley LW. Pandemic lineages of extraintestinal pathogenic Escherichia coli. Clinical Microbiology and Infection. 2014 May;20(5):380–90. doi: 10.1111/1469-0691.12646 24766445

[15] Guo S, Wakeham D, Brouwers HJM, Cobbold RN, Abraham S, Mollinger JL, et al. Human-associated fluoroquinolone-resistant Escherichia coli clonal lineages, including ST354, isolated from canine feces and extraintestinal infections in Australia. Microbes and Infection. 2015 Apr;17(4):266–74. doi: 10.1016/j.micinf.2014.12.016 25576024

[16] Nittayasut N, Yindee J, Boonkham P, Yata T, Suanpairintr N, Chanchaithong P. Multiple and High-Risk Clones of Extended-Spectrum Cephalosporin-Resistant and blaNDM-5-Harbouring Uropathogenic Escherichia coli from Cats and Dogs in Thailand. Antibiotics. 2021 Nov 10;10(11):1374. doi: 10.3390/antibiotics10111374 34827312 PMC8614778

[17] Annual report on the use of veterinary antibiotics in China (2020). Veterinary Bulletin of the Ministry of Agriculture and Rural People’s Republic of China. VOL.23. NO.9. Available from: https://www.moa.gov.cn/gk/sygb/.

[18] So JH, Kim J, Bae IK, Jeong SH, Kim SH, Lim S kyung, et al. Dissemination of multidrug-resistant Escherichia coli in Korean veterinary hospitals. Diagnostic Microbiology and Infectious Disease. 2012 Jun;73(2):195–9. doi: 10.1016/j.diagmicrobio.2012.03.010 22516765

[19] Ortega-Paredes D, Haro M, Leoro-Garzón P, Barba P, Loaiza K, Mora F, et al. Multidrug-resistant Escherichia coli isolated from canine faeces in a public park in Quito, Ecuador. Journal of Global Antimicrobial Resistance. 2019 Sep;18:263–8. doi: 10.1016/j.jgar.2019.04.002 30980959

[20] Zhu Z, Pan S, Wei B, Liu H, Zhou Z, Huang X, et al. High prevalence of multi-drug resistances and diversity of mobile genetic elements in Escherichia coli isolates from captive giant pandas. Ecotoxicology and Environmental Safety. 2020 Jul;198:110681. doi: 10.1016/j.ecoenv.2020.110681 32361498

[21] Mustapha M, Audu Y, Ezema KU, Abdulkadir JU, Lawal JR, Balami AG, et al. Antimicrobial Susceptibility Profiles of *Escherichia Coli* Isolates from Diarrheic Dogs in Maiduguri, Borno State, Nigeria. Macedonian Veterinary Review. 2021 Mar 1;44(1):47–53.

[22] Zhu Z, Jiang S, Qi M, Liu H, Zhang S, Liu H, et al. Prevalence and characterization of antibiotic resistance genes and integrons in Escherichia coli isolates from captive non-human primates of 13 zoos in China. Science of The Total Environment. 2021 Dec;798:149268. doi: 10.1016/j.scitotenv.2021.149268 34333432

[23] Saputra S, Jordan D, Mitchell T, Wong HS, Abraham RJ, Kidsley A, et al. Antimicrobial resistance in clinical Escherichia coli isolated from companion animals in Australia. Veterinary Microbiology. 2017 Nov;211:43–50. doi: 10.1016/j.vetmic.2017.09.014 29102120

[24] Scott Weese J. Antimicrobial resistance in companion animals. Anim Health Res Rev. 2008 Dec;9(2):169–76. doi: 10.1017/S1466252308001485 18983722

[25] Mustapha M, Audu Y, Ezema KU, Abdulkadir JU, Lawal JR, Balami AG, et al. Antimicrobial Susceptibility Profiles of *Escherichia Coli* Isolates from Diarrheic Dogs in Maiduguri, Borno State, Nigeria. Macedonian Veterinary Review. 2021 Mar 1;44(1):47–53.

[26] Clinical and Laboratory Standards Institute (2023). Performance standards for antimicrobial susceptibility testing, M100-33Ed, PA: Clinical and Laboratory Standards Institute.

[27] Clermont O, Bonacorsi S, Bingen E. Rapid and Simple Determination of the *Escherichia coli* Phylogenetic Group. Appl Environ Microbiol. 2000 Oct;66(10):4555–8. 11010916 10.1128/aem.66.10.4555-4558.2000 PMC92342

[28] Francisco AP, Bugalho M, Ramirez M, Carriço JA. Global optimal eBURST analysis of multilocus typing data using a graphic matroid approach. BMC Bioinformatics. 2009 Dec;10(1):152. doi: 10.1186/1471-2105-10-152 19450271 PMC2705362

[29] Tang F, Wang J, Li D, Gao S, Ren J, Ma L, et al. Comparative genomic analysis of 127 Escherichia coli strains isolated from domestic animals with diarrhea in China. BMC Genomics. 2019 Dec;20(1):212. doi: 10.1186/s12864-019-5588-2 30866824 PMC6416869

[30] Francisco AP, Bugalho M, Ramirez M, Carriço JA. Global optimal eBURST analysis of multilocus typing data using a graphic matroid approach. BMC Bioinformatics. 2009 Dec;10(1):152. doi: 10.1186/1471-2105-10-152 19450271 PMC2705362

[31] Feil EJ, Spratt BG. Recombination and the Population Structures of Bacterial Pathogens. Annu Rev Microbiol. 2001 Oct;55(1):561–90. doi: 10.1146/annurev.micro.55.1.561 11544367

[32] Algammal AM. atpD gene sequencing, multidrug resistance traits, virulence-determinants, and antimicrobial resistance genes of emerging XDR and MDR-Proteus mirabilis. Scientific Reports. 2021. doi: 10.1038/s41598-021-88861-w 33947875 PMC8096940

[33] Schmidt VM, Pinchbeck G, McIntyre KM, Nuttall T, McEwan N, Dawson S, et al. Routine antibiotic therapy in dogs increases the detection of antimicrobial-resistant faecal Escherichia coli. Journal of Antimicrobial Chemotherapy [Internet]. 2018 Sep 11 [cited 2023 Apr 22]; Available from: https://academic.oup.com/jac/advance-article/doi/10.1093/jac/dky352/5095201. 30215725 10.1093/jac/dky352

[34] Cui L, Zhao X, Li R, Han Y, Hao G, Wang G, et al. Companion Animals as Potential Reservoirs of Antibiotic Resistant Diarrheagenic Escherichia coli in Shandong, China. Antibiotics. 2022 Jun 20;11(6):828. doi: 10.3390/antibiotics11060828 35740235 PMC9220070

[35] Roberts MC, Schwarz S. Tetracycline and Phenicol Resistance Genes and Mechanisms: Importance for Agriculture, the Environment, and Humans. J Environ Qual. 2016 Mar;45(2):576–92. doi: 10.2134/jeq2015.04.0207 27065405

[36] Chopra I, Roberts M. Tetracycline Antibiotics: Mode of Action, Applications, Molecular Biology, and Epidemiology of Bacterial Resistance. Microbiol Mol Biol Rev. 2001 Jun;65(2):232–60. doi: 10.1128/MMBR.65.2.232-260.2001 11381101 PMC99026

[37] Zhang S, Chen S, Abbas M, Wang M, Jia R, Chen S, et al. High incidence of multi-drug resistance and heterogeneity of mobile genetic elements in Escherichia coli isolates from diseased ducks in Sichuan province of China. Ecotoxicology and Environmental Safety. 2021 Oct;222:112475. doi: 10.1016/j.ecoenv.2021.112475 34243112

[38] Rehman MU, Zhang H, Iqbal MK, Mehmood K, Huang S, Nabi F, et al. Antibiotic resistance, serogroups, virulence genes, and phylogenetic groups of Escherichia coli isolated from yaks with diarrhea in Qinghai Plateau, China. Gut Pathog. 2017 Dec;9(1):24. doi: 10.1186/s13099-017-0174-0 28546830 PMC5443361

[39] Peng Z, Hu Z, Li Z, Zhang X, Jia C, Li T, et al. Antimicrobial resistance and population genomics of multidrug-resistant Escherichia coli in pig farms in mainland China. Nat Commun. 2022 Mar 2;13(1):1116. doi: 10.1038/s41467-022-28750-6 35236849 PMC8891348

[40] Jiang H, Cheng H, Liang Y, Yu S, Yu T, Fang J, et al. Diverse Mobile Genetic Elements and Conjugal Transferability of Sulfonamide Resistance Genes (sul1, sul2, and sul3) in Escherichia coli Isolates From Penaeus vannamei and Pork From Large Markets in Zhejiang, China. Front Microbiol. 2019 Aug 2;10:1787. doi: 10.3389/fmicb.2019.01787 31428076 PMC6690019

[41] Zhang S, Abbas M, Rehman MU, Huang Y, Zhou R, Gong S, et al. Dissemination of antibiotic resistance genes (ARGs) via integrons in Escherichia coli: A risk to human health. Environmental Pollution. 2020 Nov;266:115260. doi: 10.1016/j.envpol.2020.115260 32717638

[42] Salinas L, Loayza F, Cárdenas P, Saraiva C, Johnson TJ, Amato H, et al. Environmental Spread of Extended Spectrum Beta-Lactamase (ESBL) Producing *Escherichia coli* and ESBL Genes among Children and Domestic Animals in Ecuador. Environ Health Perspect. 2021 Feb;129(2):027007. 33617318 10.1289/EHP7729 PMC7899495

[43] Zhou Y, Ji X, Liang B, Jiang B, Li Y, Yuan T, et al. Antimicrobial Resistance and Prevalence of Extended Spectrum β-Lactamase-Producing Escherichia coli from Dogs and Cats in Northeastern China from 2012 to 2021. Antibiotics. 2022 Oct 28;11(11):1506. 36358160 10.3390/antibiotics11111506 PMC9686880

[44] Leite-Martins LR, Mahú MIM, Costa AL, Mendes Â, Lopes E, Mendonça DMV, et al. Prevalence of antimicrobial resistance in enteric Escherichia coli from domestic pets and assessment of associated risk markers using a generalized linear mixed model. Preventive Veterinary Medicine. 2014 Nov;117(1):28–39. doi: 10.1016/j.prevetmed.2014.09.008 25294317

[45] Feng Y, Qin Z, Geng Y, Huang X, Ouyang P, Chen D, et al. Regional analysis of the characteristics and potential risks of bacterial pathogen resistance under high-pressure antibiotic application. Journal of Environmental Management. 2022 Sep;317:115481. doi: 10.1016/j.jenvman.2022.115481 35751278

[46] Fernandes V, Cunha E, Nunes T, Silva E, Tavares L, Mateus L, et al. Antimicrobial Resistance of Clinical and Commensal Escherichia coli Canine Isolates: Profile Characterization and Comparison of Antimicrobial Susceptibility Results According to Different Guidelines. Veterinary Sciences. 2022 Jun 9;9(6):284. doi: 10.3390/vetsci9060284 35737336 PMC9227429

[47] Sun Y, An Z, Chen C. Isolation and antibiotics susceptibility testing of Escherichia coli isolated from dogs in Chengdu, Si-chuan. Veterinary Orientation. 2017;(15):75–76.

[48] Wei B, Ou H, Yan W, Tian Y, Dan J, Tu R, et al. Antimicrobial resistance and integron-gene cassette detection of Escherichia coli isolates from diarrhea dogs. J. South China Agricultural University. 2018;39 (03):6–12.

[49] Saputra S, Jordan D, Mitchell T, Wong HS, Abraham RJ, Kidsley A, et al. Antimicrobial resistance in clinical Escherichia coli isolated from companion animals in Australia. Veterinary Microbiology. 2017 Nov;211:43–50. doi: 10.1016/j.vetmic.2017.09.014 29102120

[50] Poirel L, Madec JY, Lupo A, Schink AK, Kieffer N, Nordmann P, et al. Antimicrobial Resistance in *Escherichia coli*. Aarestrup FM, Schwarz S, Shen J, Cavaco L, editors. Microbiol Spectr. 2018 Jul 27;6(4):6.4.14. 10.1128/microbiolspec.arba-0026-2017 PMC11633601 30003866

[51] Yao C, Song X, Wang L, Liu B, Tian Y, Hu G. Detection of Resistance of Dog−derived Escherichia coli Isolates to Tetracy-clines. Acta Agriculturae Jiangxi. 2015;27(10):104–107.

[52] Deng X, Zheng T, Chen L, Wang M, Zhao M, Huang X, et al. Investigation on antimicrobial resistance and resistance genes of Escherichia coli from pet dogs in Fuzhou City. J. Fujian Agriculture and Forestry University (Natural Science Edition). 2022;51(06):815–821.

[53] Bevan ER, Jones AM, Hawkey PM. Global epidemiology of CTX-M β-lactamases: temporal and geographical shifts in genotype. Journal of Antimicrobial Chemotherapy. 2017 Aug 1;72(8):2145–55. 28541467 10.1093/jac/dkx146

[54] Cottell JL, Webber MA, Coldham NG, Taylor DL, Cerdeño-Tárraga AM, Hauser H, et al. Complete Sequence and Molecular Epidemiology of IncK Epidemic Plasmid Encoding *bla* _CTX-M-14_. Emerg Infect Dis. 2011 Apr;17(4):645–52. 21470454 10.3201/eid1704.101009 PMC3377399

[55] Ho PL, Yeung MK, Lo WU, Tse H, Li Z, Lai EL, et al. Predominance of pHK01-like incompatibility group FII plasmids encoding CTX-M-14 among extended-spectrum beta-lactamase–producing Escherichia coli in Hong Kong, 1996–2008. Diagnostic Microbiology and Infectious Disease. 2012 Jun;73(2):182–6. doi: 10.1016/j.diagmicrobio.2012.03.009 22521053

[56] Zhang S, Chen S, Rehman MU, Yang H, Yang Z, Wang M, et al. Distribution and association of antimicrobial resistance and virulence traits in Escherichia coli isolates from healthy waterfowls in Hainan, China. Ecotoxicology and Environmental Safety. 2021 Sep;220:112317. doi: 10.1016/j.ecoenv.2021.112317 34049228

[57] Algammal AM, El-Tarabili RM, Alfifi KJ, Al-Otaibi AS, Hashem MEA, El-Maghraby MM, et al. Virulence determinant and antimicrobial resistance traits of Emerging MDR Shiga toxigenic E. coli in diarrheic dogs. AMB Expr. 2022 Mar 17;12(1):34. doi: 10.1186/s13568-022-01371-4 35298727 PMC8931135

[58] Puño-Sarmiento J, Medeiros L, Chiconi C, Martins F, Pelayo J, Rocha S, et al. Detection of diarrheagenic Escherichia coli strains isolated from dogs and cats in Brazil. Veterinary Microbiology. 2013 Oct;166(3–4):676–80. doi: 10.1016/j.vetmic.2013.07.007 23932311

[59] Pakbin B, Brück WM, Rossen JWA. Virulence Factors of Enteric Pathogenic Escherichia coli: A Review. IJMS. 2021 Sep 14;22(18):9922. doi: 10.3390/ijms22189922 34576083 PMC8468683

[60] Čurová K, Slebodníková R, Kmeťová M, Hrabovský V, Maruniak M, Liptáková E, et al. Virulence, phylogenetic background and antimicrobial resistance in Escherichia coli associated with extraintestinal infections. Journal of Infection and Public Health. 2020 Oct;13(10):1537–43. doi: 10.1016/j.jiph.2020.06.032 32690453

[61] Kim B, Kim JH, Lee Y. Virulence Factors Associated With *Escherichia coli* Bacteremia and Urinary Tract Infection. Ann Lab Med. 2022 Mar 1;42(2):203–12. 34635614 10.3343/alm.2022.42.2.203 PMC8548248

[62] Johnson JR, Russo TA. Molecular epidemiology of extraintestinal pathogenic (uropathogenic) Escherichia coli. International Journal of Medical Microbiology. 2005 Oct;295(6–7):383–404. doi: 10.1016/j.ijmm.2005.07.005 16238015

[63] Liu X, Liu H, Li Y, Hao C. Association between virulence profile and fluoroquinolone resistance in Escherichia coli isolated from dogs and cats in China. J Infect Dev Ctries. 2017 Apr 30;11(04):306–13. doi: 10.3855/jidc.8583 28459221

[64] Rosengren LB, Waldner CL, Reid-Smith RJ. Associations between Antimicrobial Resistance Phenotypes, Antimicrobial Resistance Genes, and Virulence Genes of Fecal *Escherichia coli* Isolates from Healthy Grow-Finish Pigs. Appl Environ Microbiol. 2009 Mar;75(5):1373–80. 19139228 10.1128/AEM.01253-08 PMC2648170

[65] Venturini C, Beatson SA, Djordjevic SP, Walker MJ. Multiple antibiotic resistance gene recruitment onto the enterohemorrhagic *Escherichia coli* virulence plasmid. FASEB j. 2010 Apr;24(4):1160–6. 19917674 10.1096/fj.09-144972

[66] Sunde M, Ramstad SN, Rudi K, Porcellato D, Ravi A, Ludvigsen J, et al. Plasmid-associated antimicrobial resistance and virulence genes in Escherichia coli in a high arctic reindeer subspecies. Journal of Global Antimicrobial Resistance. 2021 Sep;26:317–22. doi: 10.1016/j.jgar.2021.06.003 34216807

[67] Poirel L, Naas T, Nordmann P. Genetic support of extended-spectrum b-lactamases. 2008; 10.1111/j.1469-0691.2007.01865.x 18154530

[68] Yazdanpour Z, Tadjrobehkar O, Shahkhah M. Significant association between genes encoding virulence factors with antibiotic resistance and phylogenetic groups in community acquired uropathogenic Escherichia coli isolates. BMC Microbiol. 2020 Dec;20(1):241. doi: 10.1186/s12866-020-01933-1 32758126 PMC7409443

[69] Rosengren LB, Waldner CL, Reid-Smith RJ. Associations between Antimicrobial Resistance Phenotypes, Antimicrobial Resistance Genes, and Virulence Genes of Fecal *Escherichia coli* Isolates from Healthy Grow-Finish Pigs. Appl Environ Microbiol. 2009 Mar;75(5):1373–80. 19139228 10.1128/AEM.01253-08 PMC2648170

[70] Osman M, Albarracin B, Altier C, Gröhn YT, Cazer C. Antimicrobial resistance trends among canine Escherichia coli isolated at a New York veterinary diagnostic laboratory between 2007 and 2020. Preventive Veterinary Medicine. 2022 Nov;208:105767. doi: 10.1016/j.prevetmed.2022.105767 36181749 PMC9703301

[71] Denamur E, Clermont O, Bonacorsi S, Gordon D. The population genetics of pathogenic Escherichia coli. Nat Rev Microbiol. 2021 Jan;19(1):37–54. doi: 10.1038/s41579-020-0416-x 32826992

[72] Manges AR, Geum HM, Guo A, Edens TJ, Fibke CD, Pitout JDD. Global Extraintestinal Pathogenic Escherichia coli (ExPEC) Lineages. Clin Microbiol Rev. 2019 Jun 19;32(3):e00135–18. doi: 10.1128/CMR.00135-18 31189557 PMC6589867

[73] Cheng P, Yang Y, Cao S, Liu H, Li X, Sun J, et al. Prevalence and Characteristic of Swine-Origin mcr-1-Positive Escherichia coli in Northeastern China. Front Microbiol. 2021 Jul 20;12:712707. doi: 10.3389/fmicb.2021.712707 34354696 PMC8329492

[74] Lu Q, Zhang W, Luo L, Wang H, Shao H, Zhang T, et al. Genetic diversity and multidrug resistance of phylogenic groups B2 and D in InPEC and ExPEC isolated from chickens in Central China. BMC Microbiol. 2022 Feb 18;22(1):60. doi: 10.1186/s12866-022-02469-2 35180845 PMC8855568

[75] Bozcal E, Eldem V, Aydemir S, Skurnik M. The relationship between phylogenetic classification, virulence and antibiotic resistance of extraintestinal pathogenic *Escherichia coli* in İzmir province, Turkey. PeerJ. 2018 Aug 24;6:e5470. 30155366 10.7717/peerj.5470 PMC6110251

[76] Oteo J, Diestra K, Juan C, Bautista V, Novais Â, Pérez-Vázquez M, et al. Extended-spectrum β-lactamase-producing Escherichia coli in Spain belong to a large variety of multilocus sequence typing types, including ST10 complex/A, ST23 complex/A and ST131/B2. International Journal of Antimicrobial Agents. 2009 Aug;34(2):173–6. 19464856 10.1016/j.ijantimicag.2009.03.006

