# Supplementary material for: Characteristics of MDR E. coli strains isolated from Pet Dogs with clinic diarrhea: A pool of antibiotic resistance genes and virulence-associated genes
Source: PLoS One. 2024 Feb 28;19(2):e0298053. doi: 10.1371/journal.pone.0298053 (PMC10901357; doi:10.1371/journal.pone.0298053)
Supplement: Attachment — Submitted filename: Response to Reviewers.docx [file pone.0298053.s008.docx]

Dear Editor:

Thank you very much for your letter and the reviewers’ comments regarding our manuscript submitted to “PLOS ONE” (Manuscript ID: PONE-D-23-30998). We have checked the article and revised it according to the comments, and carefully proof-read the manuscript. The language presentation was improved with assistance from English speakers with appropriate research background. All revisions were marked in revised manuscript with track changes.

We hope, with these modifications and improvements based on your suggestion and the reviewer’s comments, the quality of our manuscript would meet the publication standard of “PLOS ONE”. Once again, we acknowledge your comments and constructive suggestions very much, which are valuable in improving the quality of our manuscript.

We submit here the revised manuscript with track changes as well as a list of changes. Thanks again for your reconsideration of our manuscript for publication in your journal. If you have any question about this paper, please don’t hesitate to let me know.

Best wishes for you!

Sincerely yours,

Zhijun Zhong

College of Veterinary Medicine

Key Laboratory of Animal Disease and Human Health of Sichuan Province

Sichuan Agricultural University

Chengdu 611130, P. R. China

E-mail: zhongzhijun488@126.com

Response to Reviewer 1 Comments

**Response to Reviewer:**

Thank you very much for your time and thoughtful comments, many of which have been incorporated into the revised manuscript (Tracked Version). Detailed responses are as follows.

**Reviewer #1:**

General:
The study by Yuan et al. did screen 135 *E. coli* obtained from the feces of diarrheic pet dogs for their antimicrobial resistance, the antibiotic resistance genes und virulence associated genes, conducted phylogenetic grouping and multi locus sequence typing.

**Comment 1**：As the study serves the purpose characterising *E. coli* strains in order to monitor its prevalence and distribution, I strongly advise the authors to work with a writing coach or copyeditor to improve the language and readability.

**Response** 1：Thanks for your comments. We have carefully checked the English expression and made extensive English revisions under the scrutiny of native English speakers with appropriate research background.

**Comment 2**：The authors need to rerun the phylogenetic grouping and/or MLST, as it is highly unlikable that ST10 belongs to phylogroup B2. Please recheck!

**Response** 2：Thanks for your comments. We reran the phylogenetic grouping and MLST to check our results carefully, and our results were correct. Actually, this phenomenon was also observed in *E. coli* isolates from chickens in China (1) and the ExPEC isolates from humans in Turkey (2)*.* Previous studies showed that ST10 strains identified in *E. coli* from pigs in China (3) and humans in Spain (4) were mostly belonged to A or D group. These results indicates that the relationship between ST and phylogroups may vary in different *E. coli* isolated from different hosts. We have also added the corresponding discussion content for this phenomenon in our revised manuscript (Tracked Version) (Line 593-598).

**Commet 3**：The authors should reexamine the references cited in the discussion to determine whether the comparisons thus drawn are indeed based on comparable study conditions.

**Response 3**：Thanks for your comments. We have carefully checked the references to ensure that the comparisons drawn are indeed based on comparable study conditions, and we have modified the references in our revised manuscript.

**Comment 4**: The authors should reconsider, why they are drawing associations between VAGs and ARGs and explain the importance of these results.

**Response** 4：Thanks for your comments. we have added content in discussion for possible reasons of the associations between VAGs and ARGs and explanation of their importance in “discussion” section in our revised manuscript (Tracked Version) (Line 521-543): The possible reasons for drawing associations between VAGs and ARGs may be related to the co-location on the same mobile genetic elements, while negative associations indicated gene incompatibilities (5). Recent studies also showed that many ARGs were inserted into conjugative plasmids carrying VAGs (6), which also may provide conditions for drawing positive association between ARGs and VAGs.

In our study, the strongest positive association was observed between *ompT* and *sul2*, which is usually detected in plasmids (7,8), indicating the positive association between *ompT* and *sul2* may be related to the plasmid. The positive association between ARGs and VAGs implied the high risk of plasmid-mediated co-transmission of ARGs and VAGs in *E. coli*, which could accelerate the spread of VAGs and ARGs within *E. coli* populations and enhance the emergence possibility of new pathogens with increased virulence and resistance potential.

Negative associations observed in our present study may also be related to mobile genetic elements. For example, the VAG *cvaC* usually be found in virulent IncF plasmids (9), while *bla*_CTX-M_ is usually found in integron (10), the different location on mobile genetic elements may be related to the negative associations between *bla*_CTX-M_ and *cvaC* in our present study. While the specific mechanism for associations observed in our present study required more comprehensive studies to validated.

**Comment 5**: Please add limitations of the study.

**Response 5：**Thanks for your comments. We have added “Limitations” in our revised manuscript (Line 612-616): In our present study, the number of samples included was limited and the sampling site was only at our Veterinary Teaching Hospital. Future investigations should include more samples and expand sampling areas. Moreover, the use of High-throughput sequencing or Whole Genome Sequencing could provide more comprehensive information on MDR *E. coli* strains from diarrheal dogs.

**Specifics**:
Line 52-53: The authors should clarify the statement. Reference 11 was unable to find link between the examined resistance and virulence genes.

**Response:** Thanks for your comments. We have deleted the reference in our revised manuscript.

Line 58: The authors should revise the use of the term „virulent groups “ (B2 and D), as Clermont et al (Reference 19) refer to virulent extra-intestinal strains belonging to groups B2 and D. Please check throughout the manuscript.

**Response：**Thanks for your comments, we have checked the whole manuscript and changed the “virulent groups” into “virulent extraintestinal-related groups” in our revised manuscript (Tracked Version) (Line 331, 554, 558, 569, and Line 945 in legends of Fig 4).

Line 58, 216, 224, 304, 342, 360: Italics in the scientific name

**Response：**Thanks for your comments, we have modified the italics in our revised manuscript (Tracked Version) (Line 66, 253, 428, line 923 in legends of Fig 2, line 943 in legends of Fig 4).

Line 61-63: The authors should clarify the sentence to avoid confusion.

**Response：**Thanks for your comments. We have modified the sentence in our revised manuscript (Tracked Version) (Line 68-75): Recent studies have identified a high population diversity of STs which related to zoonotic or pathotype in *E. coli* isolates from pet dogs, such as ST354, ST393, and ST457 *E. coli* were observed in companion animals from Australia. And the high-risk ExPEC clones associated with humans and multidrug resistance, including sequence type (ST) 38, ST131, ST224, ST167, ST354, ST410, ST617 and ST648, have been identified in cats and dogs in Thailand which suggested there may be clonal dissemination between pets and humans.

Line 70-71: Bulletin of the Ministry of Agriculture and Rural People’s Republic of China, 2020). (27). Please check punctuation.

**Response：**Thanks for your comments, we deleted the extra punctuation in our revised manuscript (Tracked Version) (Line 80-81).

Line 76: Please change diarrheal in diarrheal disease or diarrhea.

**Response：**Thanks for your comments, we have changed diarrheal into diarrhea in our revised manuscript (Tracked Version) (Line 96).

Line 99: Please change 16 S rRNA into 16S rRNA and Primer:5’-G to Primer: 5’-G

**Response：**Thanks for your comments, we have change 16 S rRNA into 16S rRNA and Primer:5’-G to Primer: 5’-G in our revised manuscript (Line 116-117).

Line 114, 173, 332, 347, 349: Please use the correct abbreviation for SXT.

**Response：**Thanks for your comments, we have checked the whole manuscript and modified the abbreviation for SXT in our revised manuscript (Tracked Version) (Line 142, 207, 382, and 409).

Line 116: Since there are no breakpoints for tobramycin, ciprofloxacin, cefuroxime,
cefotaxime, cefepime, cefoxitin, aztreonam in CLSI Vet06 2023, the authors should clarify how they obtained the breakpoints.

**Response：**Thanks for your comments. The references insert here is wrong. The correct referenced standard for our present study was the CLSI 2023 (Performance standards for antimicrobial susceptibility testing, M100-Ed33), and the Interpretive Categories and Zone Diameter Breakpoints for the antimicrobial agents which we tested are showed in Table S6. We have revised this reference in our revised manuscript.

Line 138-140: The authors should explain why they used the protocol of Clermont from 2000 which distinguishes phylogroups A, B1, B2, and D instead of the new protocol from 2013 which enables an E. coli isolate to be assigned to one of the eight phylogroups (A, B1, B2, C, D, E, F and Escherichia cryptic clade I).

**Response：**Thanks for your comments. Firstly, the protocol of Clermont was still widespread used in recent studies of *E. coli* isolates from diarrheal dogs (11,12). Secondly, phylogenetic protocol from Clermont has shown that virulent extraintestinal strains mainly belong to groups B2 and D, whereas strains of groups A and B1 are usually devoid of extraintestinal virulence factors, indicating a link between phylogroups and extraintestinal virulence (13,14). Our present study focuses on the antibiotic resistance characteristics of MDR *E. coli* from diarrheal dogs and preliminary analysis of the phylogroups and MLST for MDR *E. coli*. Our results showed the most common ST was ST10 and ST10 strains mostly belonged to B2 groups. As the reviewer suggests that the new protocol from 2013 can get more comprehensive phylogroups, we will consider using the new protocol in our future research.

Line 177-178: Please change Results s howed to Results showed

**Response：**Thanks for your comments, the “showed” has been modified to “revealing “in our revised manuscript (Tracked Version) (Line 212).

Line 179-180: Please change were o bserved to were observed

**Response：**Thanks for your comments, we have changed o bserved into observed in our revised manuscript (Tracked Version) (Line 213).

Figure 1.B: Please change the image in favor of clarity and quality. For example, summarize combinations that occur only once and enlarge the remaining that occur multiple times.

**Response：**Thanks for your comments. We have added red boxes in the figure to highlight the prevalent resistant-phenotypes patterns (occur three times) in our revised Figure 1. And the other combinations (without red box) occur only once or twice. The corresponding explanation for the red boxes has been added to the figure legends of Fig 1.B (Line 919-921).

Line 186: Please change 0 R to 0R according to figure 1.A. Please revise the language in the following sentences
Table 1: Please change“Antibiotics”in the top heading to “Antibiotic”. Please insert a space between number of isolates and percentage in the first column. Please change 1rd/2th to 1st/2nd. As the interpretation of zone diameter or MIC changes with revised breakpoints every few years, a table with the results of the zone diameters should be added as a supplementary figure.

**Response：**Thanks for your comments. We have change 0 R to 0R according to figure 1.A (Line 914). For Table 1, we have changed“Antibiotics”in the top heading to “Antibiotic”, inserted a space between number of isolates and percentage in the first column, and change 1rd/2th to 1st/2nd (Line 228). Moreover, the zone diameter breakpoints for the antimicrobial susceptibility testing in our present study are showed in Table S6.

Line 201, 212, 206, 207, 373: Please check gene names.

**Response：**Thanks for your comments, we have modified the gene names in our revised manuscript (Tracked Version) (Line 235, 248, 239, 239, 443).

Line 218-220: The authors should clarify the sentence to avoid confusion.

**Response：**Thanks for your comments, we have modified the sentence to avoid confusion in our revised manuscript (Tracked Version) (Line 255-257): The average number of VAGs carried by per DEC-related strain (8.36) was significantly higher than that of DEC-unrelated VAGs strains (5.56) (*P* < 0.01).

Table 2: Please remove the % from the percentage of resistance to beta-lactams. Your results of tetracyclines suggest you found strains which exhibited resistance to doxycycline and at the same time susceptibility to tetracycline. Did you recheck these?

**Response：**Thanks for your comments, we have removed the % from the percentage of resistance to beta-lactams in our revised manuscript (Tracked Version) (Table 2). For the phenomenon of strains showing resistance to doxycycline and at the same time susceptible to tetracycline, we checked the detailed results of AMR, there are four strains resistant to doxycycline and at the same time susceptible to tetracycline. Moreover, the similar phenomenon was observed in our previous study for *E. coli* from captive non-human primates (15) and *E. coli* from captive giant pandas (16). While the reason for this phenomenon is unclear and needs to be investigated in further experiments.

Line 261: You write “The other 2 pairs (blaCTX-M/tsh, blaCTX-M/iss) were negative association (*r* < 0, *P* < 0.05)”. In Figure 3 it shows a negative association with cvaC instead of iss. Please check.

**Response：**Thanks for your comments, we have changed it in our revised manuscript (Line 247): The other 2 pairs (*bla*_CTX-M_/*tsh*, *bla*_CTX-M_/*cvaC*) were negative association.

Line 284: Please check sentence with keeping in mind that no strains belonged to phylogroup A.

**Response：**Thanks for your comments, we have rewritten in our revised manuscript (Tracked Version) (Line 325).

Line 286-288: Please explain the marking representing significance in Figure 1.B.
**Response：**Thanks for your comments, we have added the sentence of “Marking * represents significant difference, *, *P* < 0.05; **, *P* < 0.01; ***, *P* < 0.001” in revised legends of Figure 4.B (Line 948-949).

Figure 4.C and 4.D: Please clarify the meaning of the different coloured outlines of the circles.

**Response：**Thanks for your comments. The yellow-green outlines of the circles represent the ST is the founder ST of one clonal complex (CC), and the other STs (with purple outlines of the circles) of the CC are derived from the founder ST with two allelic differences. We have added the meaning of the different colored outlines of the circles in our revised Figure legends (Line 954-957).

Figure 4.E: Please clarify on which data the tree is based.

**Response：**Thanks for your comments. The Heatmap was performed by “pheatmap” in R-studio based on the distribution of phylogenetic groups and MLST for MDR strains in our study. The distribution of phylogenetic groups and MLST was transformed to “0” or “1”. The partial data of the transformation results are shown in the table below. For example, data for CD01 in the table below means the strain belonged to B2 group and ST10, CD02 in the table below means the strain belonged to D group and ST58.

| NAME | B1 | B2 | D | ST10 | ST58 | ST361 | ST1276 |
| --- | --- | --- | --- | --- | --- | --- | --- |
| CD01 | 0 | 1 | 0 | 1 | 0 | 0 | 0 |
| CD02 | 0 | 0 | 1 | 0 | 1 | 0 | 0 |
| CD03 | 0 | 1 | 0 | 0 | 0 | 1 | 0 |
| CD04 | 0 | 1 | 0 | 1 | 0 | 0 | 0 |
| CD05 | 0 | 0 | 1 | 0 | 0 | 0 | 1 |
| CD06 | 0 | 1 | 0 | 0 | 0 | 0 | 0 |

……

Line 368-369: You stated you found blaTEM-1, blaTEM-135, and blaTEM-176. Neither of these is a ESBL-encoding gene. Please rephrase the sentence accordingly.

**Response：**Thanks for your comments. We have checked the whole manuscript and changed the ESBL-encoding gene to β-lactam resistance genes in order to avoid confusion (Line 439).

Line379-380: Please add in Material and Methods, how you located the blaCTX-M-14 on the plasmid and present the data in results.

**Response：**Thanks for your comments. In our present study, we did not investigate the location of *bla*_CTX-M-14_. This sentence was proposed based on the references which provided to indicate the potential transmission risk of *bla*_CTX-M-14_ strains. And the references have been added at the end of the sentence (Line 449-450).

Line 383-388: The authors use very general terms in the two sentences. Please clarify, what “paying more attention” means. Please explain in more detail what you mean by “diversity and evolution” and the implications of that, or refrain from using the sentence.

**Response：**Thanks for your comments. In our present study, the high prevalence of *bla*_CTX-M-14_-positive and *bla*_CTX-M-55_-positive strains was observed, which are considered as high-risk strains for transmission between different hosts, thus, “paying more attention” means that more studies should focus on the capability of clonal transmission between different hosts for these strains in the future. We have added the corresponding content in our revised manuscript (Tracked Version) (Line 458-460).

Moreover, the other uncommon variants of *bla*_CTX-M_ were not the focus of our discussion, so we have deleted the “diversity and evolution” in our revised manuscript.

Line 399-400: The authors are repeating the sentence from 391-392. Please amend.

**Response：**Thanks for your comments. We have deleted the repeating sentence in our revised manuscript.

Line 405-406: The authors speak in very general terms. The manuscript would improve by the use of examples.

**Response：**Thanks for your comments. We have modified the sentence in our revised manuscript (Tracked Version) (Line 480-485): The similar phenomenon was also found in *E. coli* from captive non-human primates and waterfowl in China which indicated the different expression of ARGs, such as the abnormal expression of ARGs and the expression of ARGs has not reached that level which can activate the antibiotic resistance, may be a possible reason the incomplete consistency between AMR and ARGs.

Line 412: Please cross-reference the percentages presented with the sources.

**Response：**Thanks for your comments. We have checked the percentages in reference and changed the sentence to “the ranging from 0.6% to 81.6% in Shaanxi and 2.53% to 87.34% in Shandong, respectively” in our revised manuscript (Tracked Version) (Line 501).

Line 412-414: As in the cited study a different set of virulence genes was tested, a comparison with the results of the study appears difficult. Please revise or delete.

**Response：**Thanks for your comments. We have deleted the sentence in our revised manuscript.

Line 416-419: Since the study does not provide data on the prevalence of combining VAGs separated into categories of ExPEC-related and DEC-related VAGs, it is difficult to follow the argument of the sentence.

**Response：**Thanks for your comments. In our present study, we separated the VAGs into categories of ExPEC-related VAGs (17–20) and DEC-related VAGs (21–23) according to previous studies. And 75.68% (56/74) of the strains carried at least one DEC-related VAG, which also carried at least one ExPEC-related VAG. We have added the corresponding data of strains combining ExPEC-related and DEC-related VAGs simultaneously in the “Results” section of our revised manuscript (Tracked Version) (Line 254-255).

Line: 428-429: The authors repeat the results already given in discussion. Please delete.

**Response：**Thanks for your comments. We have deleted the repeating sentence in our revised manuscript.

Line 431-434: The authors explain the possible reason for the positive correlation between sul2 and ompT very well. Is there a similar rationale for the other two positive pairs? Is anything known about possible reasons for the negative association pairs?

**Response** 4：Thanks for your comments. We have added corresponding content in discussion for the possible reasons for the other two positive pairs and negative association pairs in our revise manuscript (Tracked Version) (Line 531-543): Similarly, the location of *aacC*, which usually was detected in the variable region of integron (24), may be related to the other two positive associations (*aacC2*/*ompT*, *aacC4*/*astA*) observed in our present study.

For the negative associations, the gene incompatibilities may be one of possible reasons according to previous researches (5,25). For example, the VAG *cvaC* usually be found in virulent IncF plasmids (9), while *bla*_CTX-M_ is usually found in integron (10), the different location on mobile genetic elements may be related to the reason for negative association between *bla*_CTX-M_ and *cvaC* observed in our present study*.* While the specific mechanism for the associations observed in our present study required more comprehensive studies to validate.

Line 442-443: Please clarify the sentence to avoid the reader’s confusion.

**Response：**Thanks for your comments. We have modified the sentence in our revised manuscript (Tracked Version) (Line 556-559): our present results showed a high prevalence of strains which belonged to virulent extraintestinal-related groups (B2 and D) were detected among MDR *E. coli* from pet dogs.

Line 448-449: Please clarify the sentence to avoid the reader’s confusion.

Response：Thanks for your comments. We have modified the sentence to “These phenomena indicated that the antimicrobial resistance may increase the possibility for carrying more virulent factor (for example, the number of VAGs carried) in *E. coli* strains” in our revised manuscript (Tracked Version) (Line 570-572). Actually, the similar phenomenon was found in previous study, which indicated that the probability of identifying virulence factor, such as STb, VT2, AIDA, was higher in strains carrying *dhfrXIII*, *dhfrI*, and *tetB*, respectively (5).

Line 452: According to the reference, ST131 has been identified the most common sequence type in ExPEC. Please rephrase.

**Response：**Thanks for your comments. We have modified the sentence to “which has been identified as the most common ST in ExPEC, covering all geographic regions” in our revised manuscript (Tracked Version) (Line 583).

Line 455: The reader might wonder as ST10 is the most prevalent sequence type in the study, if there is anything known about its distribution?

**Response：**Thanks for your comments. Actually, ST10 also was widely detected in *E. coli* from other animals in China, such as pigs (3,26) and chickens (1), indicating that the ST10 may be the prevalent ST in *E. coli* strains from animals in China. And we have also added the corresponding content in our revised manuscript (Tracked Version) (Line 591-593).

Line 462-463: Please specify.

**Response：**Thanks for your comments. We have changed the sentence to “Seventy-four MDR strains were observed among 135 *E. coli* isolates from pet dogs, of which 12 ARGs and 14 VAGs were detected, implying that the *E. coli* isolates can be considered as a pool of ARGs and VAGs” in our revised manuscript (Tracked Version) (Line 601-604).

Response to Reviewer 2 Comments

**Response to Reviewer:**

Thank you very much for your time and thoughtful comments, many of which have been incorporated into the revised manuscript (Tracked Version). Detailed responses are as follows.

**Reviewer #2:**

(1) Abstract: So far I know, any abstract has four essential parts with few additional or optional parts, such as objective(s), methodology, findings, and concluding remarks as essential parts and background, limitations, future indication etc could be present as additional portion(s). In this abstract, the objectives and methodology were not properly written, even absent. The abstract should be re-written properly, concisely and with proper structures.

**Response 1：**Thanks for your comments. As the reviewer suggested, we have modified the “Abstract” in our revised manuscript (Tracked Version) (Line 15-41).

(2) Introduction: In my opinion, research gaps, justification and novelty of the present research were not mentioned or presented properly.

**Response 2：**Thanks for your comments. Recently, increasing studies have focused on antimicrobial resistant *E. coli* from companion animals, indicating that the companion animals are reservoirs of MDR strains with a high risk of transmission between dogs and humans (27). However, the research on MDR strains from companion animals in China is limited, especially in Sichuan Province, which is considered to be a major province of pets keeping in western China (28). In our present study, we not only analyze the antibiotic resistance of *E. coli* isolated from pet dogs, but also pay more attention to the ARGs, VAGs and population structure (ST and phylogenetic groups) for MDR strains to evaluate the potential threat. Moreover, more and more studies have observed the association which existed in between ARGs and VAGs in *E. coli* from animals, such as pandas (29) and waterfowl (19), but studies for the association between ARGs and VAGs of *E. coli* from pet dogs was limited, we further analyzed the association between ARGs and VAGs in MDR strains. Above all, our research helps to better understand the characteristics of MDR strains from pet dogs and fills the research gaps of MDR *E. coli* from pet dogs in Sichuan Province. And we have modified the “Introduction” section in our revised manuscript (Tracked Version) (Line 46-90).

(3) Methodology:
(A) Sample size could be larger, and could be determined using formula. However, the less number of samples could be explained properly in the 2.1 section.

**Response 3A:** Thanks for your comments. Actually, we collected about 250 fecal samples in the initial phase of the present study, while some of the samples did not meet the sampling standards for “Samples were excluded if the pet dogs were prescribed antimicrobial therapy or veterinary admission within the previous 3 months” in our present study. The sampling standards were also shown in 2.1 section (Line 83-84). Thus, only 185 fecal samples were ultimately included. We have added the explanation for limited number of samples in the “Limitations” section in our revised manuscript (Tracked Version) (Line 612-616).

(B) Line 77: why this findings unnecessarily came here?

**Response 3B:** Thanks for your comments. We have deleted this finding in 2.1 section and added it to “Results” section in our revised manuscript (Tracked Version) (Line 199-200).

Line 80-81: Could you please provide the permission number?

**Response:** Thanks for your comments. The permission number is DYY-2020303164, and we have added it in our revised manuscript (Tracked Version) (Line 100).

(C) strain isolation portion (section 2.2) was made unnecessarily complex.

**Response 3C:** Thanks for your comments. We have simplified the section 2.2 in our revised manuscript (Tracked Version) (Line 111-127): The isolation and identification of *E. coli* was performed as previous studies described. Fecal samples were enriched in Luria-Bertani (LB) broth at 37 °C, 120 r/min in a shaking incubator for 24 h. All isolates were identified presumptively by using phenotypic methods, including Gram staining, MacConkey agar growth and Eosin Methylene Blue agar growth. We further used 16S rDNA sequences (Primer: 5’-GAGTTTGATCCTGGCTCAG-3’; 5’-AGAAAGGAGGTGATCCAGCC-3’) for final identification of *E. coli*. The confirmed isolates were stored in Luria-Bertani (LB) broth containing 50% glycerol at −20 °C for further analysis.

(D) Line 113-115: these information could be added in discussion.

**Response 3D:** Thanks for your comments. Actually, the information is the criteria for our selection of antimicrobial agents for susceptibility testing in our present study, it is more scientific to place the information in “2.3” section, and we have modified the sentence to“For the 16 antimicrobial agents, the CN and TOB in aminoglycosides, DOX in tetracyclines, KZ, CTX, AMP, AMC in β-lactams and CIP in quinolones were used in pet animals at the location of our present study. The other antimicrobial agents (TET, C, CXM, FEP, ATM, IPM, FOX and SXT) have been found with *E. coli* resistance in dogs according to previous studies” in our revised manuscript (Tracked Version) (Line 139-143).

(E) Line 124-125: please rephrase the sentence.

**Response 3E:** Thanks for your comments. We have rephrased the sentence to “Primers of 23 ARGs (including 5 *bla*_CTX-M_ alleles for group1, 2, 8, 9 and 25) in 6 categories and 25 VAGs in 5 categories were synthesized by Huada Gene Technology Co. Ltd (Shenzhen, China), and the PCR primer and conditions for the ARGs and VAGs which been chosen in our present study were showed in S1 Table and S2 Table, respectively” in our revised manuscript (Line 128-131).

(F) Line 127-130: Could be added to the discussion.

**Response 3F:** Thanks for your comments. We have moved the sentence to “Discussion” section in our revised manuscript (Tracked Version) (Line 492-496): In our present study, 25 VAGs were chosen to detected according previous studies, among which 12 VAGs (*eae, papA, bfpA, aggR, pic, astA, ipaH, stx1, stx2, elt, esta and estb*) are related to Diarrhoeagenic *Escherichia coli* (DEC), and 13 VAGs (*fyuA, iroN, sitA, iucD, Irp2, fimC, tsh, vat, ompT, cvaC, iss, hlyF, hlyA*) are related to Extraintestinal *Escherichia coli* (ExPEC).

(G) The 2.4 section was not described properly and scientifically. Supplementary Table information should be mentioned.

**Response 3G:** Thanks for your comments. We have modified the 2.4 section in our revised manuscript (Tracked Version) (Line 149-168).

(4) Results: poorly represented.

(A) for determination of ARGs and AMR or VAGs association, in my opinion, all the E. coli isolates should be taken into consideration. Only MDR or resistant isolates can not give any conclusive data. Moreover, for this purpose, the sample size should be larger than the present research work.

**Response 4A:** Thanks for your comments. In recent years, the presence of MDR *E. coli* in companion animals, such as pet dogs, undoubtedly raises more concerns for both dogs and humans due to the high antimicrobial resistance (AMR) and the capability of MDR strains to carry various antibiotic resistance genes (ARGs) (30–32). Thus, we focused on the MDR *E. coli* strains from diarrheal dogs, which pose greater healthy threat to humans, to investigate the associations between ARGs and AMR or VAGs.

(B) Phylogroup and ST distribution was not properly depicted.

**Response 4B:** Thanks for your comments. We have modified the description for Phylogroup and ST distribution in our revised manuscript (Tracked Version) (Line 319-350).

(C) Text was elaborately presented, so that the table 1 and 2 were unnecessarily added.

**Response 4C:** Thanks for your comments. The text in results section just displayed the main results of our study. Table 1 and 2 show the whole details of AMRs and ARGs in MDR *E. coli* strains which give more comprehensive and intuitive data for readers to understand.

(5) Discussion: poorly written. repetition of results came many times. Exact platform was not made.

**Response 5:** Thanks for your comments. We have substantially revised our discussion and deleted the repetition of results in our revised manuscript.

(6) Conclusions: did not reflect the research outcome properly.

**Response 6:** Thanks for your comments. We have modified the conclusion in our revised manuscript (Tracked Version) (Line 601-609).

(7) References: huge reference list. should be restricted within 50-55. better less than 40.

**Response 7:** Thanks for your comments. We have tried our best to simplify the reference list which is incited in our manuscript, while we have added a lot of discussion content based on comments of the reviewers, which also requires necessary references to support. Thus, the reference list is limited to 76.

(8) Others: Grammatical errors and poor English in many places of the manuscript.
Moreover, I could not find any new information from this research works. Though have few new ideas. However, following published articles could be mentioned as justification or research gaps...

**Response 8:** Thanks for your comments. We have carefully checked the English expression and make extensive English revisions under the scrutiny of native English speakers with appropriate research background.

Actually, there are many studies on resistant *E. coli* which indicated that high resistance was occurred in *E. coli* isolates from companion animals due to the abuse of antibiotics. However, only limited studies specifically focused on MDR strains from pets in China, especially in Sichuan province which is considered as a major province for keeping pets in Southwest China. In our present study, we analyzed the MDR *E. coli* isolates from diarrheal dogs by detecting ARGs, VAGs and population structure, the results revealed that high diversity of ARGs and VAGs were observed in MDR canine *E. coli,* and potential pathogenic groups have advantage in *E. coli* isolates, indicating that the diarrheal dogs could be considered as potential reservoirs of resistant and pathogenic *E. coli.* These findings may have important implications for preliminary understanding of the characteristics of MDR *E. coli* from diarrheal dogs and for evaluating the potential threat.

Moreover, thank you for providing these references. We have taken inspiration from them and made major revisions in our manuscript. In the future investigation of our team, we will refer the innovation of these researches to obtain more comprehensive information of MDR strains from companion animals and further take effective measures to control the increasing of MDR strains.

https://doi.org/10.1128%2FJCM.40.10.3586-3595.2002
Sanchez S, McCrackin Stevenson MA, Hudson CR, Maier M, Buffington T, Dam Q, Maurer JJ. Characterization of multidrug-resistant Escherichia coli isolates associated with nosocomial infections in dogs. Journal of Clinical Microbiology. 2002 Oct;40(10):3586-95.

https://doi.org/10.5455/javar.2020.g466IF: 1.4 Deb, P., Das, T., Nath, C., Ahad, A., & Chakraborty, P. (2020). Isolation of multidrug-resistant Escherichia coli, Staphylococcus spp., and Streptococcus spp. from dogs in Chattogram Metropolitan Area, Bangladesh. Journal of Advanced Veterinary and Animal Research, 7(4), 669-677

https://doi.org/10.1128/AEM.00599-11IF: 4.4 Q2 Karczmarczyk M, Abbott Y, Walsh C, Leonard N, Fanning S. Characterization of multidrug-resistant Escherichia coli isolates from animals presenting at a university veterinary hospital. Applied and Environmental Microbiology. 2011 Oct 15;77(20):7104-12.

https://doi.org/10.3389/fvets.2023.1104812IF: 3.2 Q1 Tong YC, Zhang YN, Li PC, Cao YL, Ding DZ, Yang Y, Lin QY, Gao YN, Sun SQ, Fan YP, Liu YQ. Detection of antibiotic-resistant canine origin Escherichia coli and the synergistic effect of magnolol in reducing the resistance of multidrug-resistant Escherichia coli. Frontiers in Veterinary Science. 2023 Mar 15;10:1104812.

https://doi.org/10.1016/j.prevetmed.2022.105767IF: 2.6 Q1 Osman M, Albarracin B, Altier C, Gröhn YT, Cazer C. Antimicrobial resistance trends among canine Escherichia coli isolated at a New York veterinary diagnostic laboratory between 2007 and 2020. Preventive Veterinary Medicine. 2022 Nov 1;208:105767.

Response to Reviewer 3 Comments

**Response to Reviewer:**

Thank you very much for your time and thoughtful comments, many of which have been incorporated into the revised manuscript (Tracked Version). Detailed responses are as follows.

**Reviewer #3:**

The manuscript described isolation of MDR E. coli strains from pet dogs carrying different antimicrobial resistance genes. The isolates were mostly associated with virulent phylogroups (B2 and D) and ST10 clonal lineage. The objectives are interesting and the methodology is standard. Language is poor and the manuscript is full of typing, grammatical and syntax errors which should be rectified by a well-versed English speaker. Most of the findings lack proper justification in the discussion section. Consequently, the conclusions are not supported with the findings.
**Response:** Thanks for your comments. We have carefully checked the English expression and make extensive English revisions under the scrutiny of native English speakers with appropriate research background. And we have substantially modified the results, discussion and conclusion in our revised manuscript.

Following issues should be replied point-wise.

Line 28. blaTEM-1 cannot be considered as ESBL…if it is necessary to describe TEM-1, please replace the ‘ESBL’ with ‘beta-lactamase’ throughout the manuscript

**Response:** Thanks for your comments. We have replaced the ‘ESBL’ with ‘beta-lactamase’ throughout the revised manuscript.

Line 76. ‘clinical diarrheal’ should be replaced with ‘clinical diarrhea’

**Response:** Thanks for your comments. We have replaced the ‘clinical diarrheal’ with ‘clinical diarrhea’ in the revised manuscript (Tracked Version) (Line 96).

Line 81. ‘animal ethics committee’ should be added

**Response:** Thanks for your comments. We have added the animal ethics committee and permission number in our revised manuscript (Tracked Version) (Line 101-102): The study was permitted by committee of Sichuan agricultural university (Permission number: DYY-2020303164) and the Sichuan Agricultural University Animal Ethical and Welfare Committee (Permission number: 20210268).

Line 82. ‘professional veterinaries’ should be replaced with ‘professional veterinarians’

**Response:** Thanks for your comments. We have replaced the ‘professional veterinaries’ with ‘professional veterinarians’ in our revised manuscript (Tracked Version) (Line 103).

Line 87. ‘Eppendorf (EP) tubes’ should be replaced with ‘micro centrifuge tubes (Eppendorf)’

**Response:** Thanks for your comments. We have replaced the “Eppendorf (EP) tubes” with “micro centrifuge tubes (Eppendorf)” in our revised manuscript (Tracked Version) (Line 109).

Line 87. Clinical history of the pet dogs is missing, age/breed/sex of the diarrhoeic dogs, types of diarrhoea (mucoid/ bloody etc.), description of other clinical symptoms like fever, anorexia, vomition etc. should be added

**Response:** Thanks for your comments. We have added the clinical history of the pet dogs which been involved (which successfully isolated *E. coli* strains) in our present study as Table S7 in our revised supporting information files.

Line 91. ‘Fecal samples were enriched in Luria-Bertani (LB) broth at 37 °C, 120 r/min for 24 h’…if the authors mean to describe about incubation in shaking incubator, that should be indicated
**Response:** Thanks for your comments. We have added “in a shaking incubator” in our revised manuscript (Tracked Version) (Line 114).

Line 94. ‘singe clone’ ??
**Response:** Thanks for your comments. This is spelling mistake, we have modified the 2.2 section, and the sentence have been deleted in our revised manuscript.

Line 113. I agree with the authors that the canine E. coli isolates earlier showed resistance against the antibiotic discs selected in the present study. However, are these antibiotics commonly used in pet/companion animals in the study location? Did you consider to go through the prescriptions or did you conduct any qualitative survey to know the antibiotic usage pattern in local canine population?

**Response:** Thanks for your comments. Among the 16 selected antimicrobial agents, CN and TOB in aminoglycosides, DOX in tetracyclines, KZ, CTX, AMP, AMC in β-lactams, and CIP in quinolones were used at the study location according to the prescriptions of veterinarians at our Veterinary Teaching Hospital. The other antimicrobial (TET, C, CXM, FEP, ATM, IPM, FOX and SXT) have been found with *E. coli* resistance in dogs according to previous studies (33–36). According to previous studies, frequent use of antimicrobial agents in clinics may be responsible for the high resistance (37,38), such as the highest resistance rate to AMP observed in present study may related to the widely use of β-lactam antibiotics in our study location. We have added the corresponding discussion in our revised manuscript (Tracked Version) (Line 410-414).

Line 135. After BLASTing did you submit the sequences into genbank? If so, please indicate the accession numbers

**Response**: Thanks for your comments. Our purpose of the present study is to detect the presence of the corresponding genes in the *E. coli* isolates. We did not upload the relevant sequence to public database due to the large amount of data. For the confirmation of each ARG, VAG, and genes related to phylogroups and MLST, each PCR sample was subjected to electrophoresis, and the positive samples were Sanger sequenced in both directions. The DNA sequence data were confirmed via the GenBank online BLAST software. Corresponding contents have been added in the revised manuscript (Tracked Version) (Line 165 to 168 and 179-181).

Line 334. Did you find use of tetracycline as growth promoter in the study location? Moreover, how did you correlate growth promotional use of tetracyclines with higher occurrence of TET-resistant isolates in canine population?
**Response:** Thanks for your comments. Tetracycline was not used as growth promoter in the study location recently. TET has been used for treating animal infection disease and also been used as “growth promoters” or “feed efficiency products” for a long time, which may activate the resistance of TET in *E. coli* from animals*.* Moreover, once antimicrobial resistance in *E. coli* has been activated, it will sustain for a long time and not easy to eliminate (39), and the antibiotics resistance genes which encode AMR also can transmission through environment and mobile genetic elements (15,19,40), which may be one possible reason for high TET-resistance in *E. coli* in our present study, even though tetracycline has not been used in the location. Moreover, our previous research has also found a similar phenomenon for high TET-resistance in *E. coli* isolated from captive non-human primates which did not use TET (15).

Line 345. ‘Those phenomena indicated that AMR sustain for a long time and not easy to eliminate, once AMR is activated’ ….I found the sentence ambiguous which should be modified or deleted. It seems that implementation of antibiotic use restriction in the study location is not very effective or there are resistant strains circulating in the canine population since long before the imposition of antibiotic usage restriction and the strains are difficult to eliminate currently.
**Response:** Thanks for your comments. We have modified the sentence to “Once the antimicrobial-resistant *E. coli* occurs, it exists for a long time due to hard elimination” in our revised manuscript (Tracked Version) (Line 388-389). Actually, we agree with your view about the implementation of antibiotic use restriction in the study location is not very effective, it may also be one of important reasons for high antibiotics resistance in *E. coli* isolates form dogs, thus, we added corresponding content in our revised manuscript (Tracked Version) (Line 425-427): Overall, resistant strains may have been circulating in the dog population long before the antibiotic usage restriction were introduced, and AMR strains are currently difficult to eliminate. The high detection rates of MDR *E. coli* in our study implied that more effective measures should be taken to control the occurrence and spread of MDR *E. coli* from companion animals.

Line 348. ‘Frequent use of β-lactams and sulfonamides antimicrobial agents in clinic…’ another speculation, it should be justified with the screening of prescriptions in the clinics used in the present study.

**Response:** Thanks for your comments. “Frequent use of β-lactams and sulfonamides antimicrobial agents in clinic may be responsible for the high resistance to AMP and SXT” was speculated according to the references which indicated the β-lactams, sulfonamides were China’s most animal-used antimicrobials (38,41). Actually, the β-lactams antimicrobial agents, such as ampicillin, amoxicillin/clavulanic acid et al., and have been used frequently in the location of our present study. We have also added the content in our revised manuscript (Tracked Version) (Line 410-414).

References

1.	Lu Q, Zhang W, Luo L, Wang H, Shao H, Zhang T, et al. Genetic diversity and multidrug resistance of phylogenic groups B2 and D in InPEC and ExPEC isolated from chickens in Central China. BMC Microbiol. 2022 Feb 18;22(1):60.

2.	Bozcal E, Eldem V, Aydemir S, Skurnik M. The relationship between phylogenetic classification, virulence and antibiotic resistance of extraintestinal pathogenic *Escherichia coli* in İzmir province, Turkey. PeerJ. 2018 Aug 24;6:e5470.

3.	Cheng P, Yang Y, Cao S, Liu H, Li X, Sun J, et al. Prevalence and Characteristic of Swine-Origin mcr-1-Positive Escherichia coli in Northeastern China. Front Microbiol. 2021 Jul 20;12:712707.

4.	Oteo J, Diestra K, Juan C, Bautista V, Novais Â, Pérez-Vázquez M, et al. Extended-spectrum β-lactamase-producing Escherichia coli in Spain belong to a large variety of multilocus sequence typing types, including ST10 complex/A, ST23 complex/A and ST131/B2. Int J Antimicrob Agents. 2009 Aug;34(2):173–6.

5.	Rosengren LB, Waldner CL, Reid-Smith RJ. Associations between Antimicrobial Resistance Phenotypes, Antimicrobial Resistance Genes, and Virulence Genes of Fecal *Escherichia coli* Isolates from Healthy Grow-Finish Pigs. Appl Environ Microbiol. 2009 Mar;75(5):1373–80.

6.	Venturini C, Beatson SA, Djordjevic SP, Walker MJ. Multiple antibiotic resistance gene recruitment onto the enterohemorrhagic *Escherichia coli* virulence plasmid. FASEB J. 2010 Apr;24(4):1160–6.

7.	Ben Said L, Jouini A, Fliss I, Torres C, Klibi N. Antimicrobial resistance genes and virulence gene encoding intimin in Escherichia coli and Enterococcus isolated from wild rabbits (Oryctolagus cuniculus) in Tunisia. Acta Vet Hung. 2019 Dec;67(4):477–88.

8.	Schmidt VM, Pinchbeck GL, Nuttall T, McEwan N, Dawson S, Williams NJ. Antimicrobial resistance risk factors and characterisation of faecal E. coli isolated from healthy Labrador retrievers in the United Kingdom. Prev Vet Med. 2015 Apr;119(1–2):31–40.

9.	Sunde M, Ramstad SN, Rudi K, Porcellato D, Ravi A, Ludvigsen J, et al. Plasmid-associated antimicrobial resistance and virulence genes in Escherichia coli in a high arctic reindeer subspecies. J Glob Antimicrob Resist. 2021 Sep;26:317–22.

10.	Poirel L, Naas T, Nordmann P. Genetic support of extended-spectrum b-lactamases. 2008;

11.	Karahutová L, Mandelík R, Bujňáková D. Antibiotic Resistant and Biofilm-Associated Escherichia coli Isolates from Diarrheic and Healthy Dogs. Microorganisms. 2021 Jun 19;9(6):1334.

12.	Vega-Manriquez XD, Ubiarco-López A, Verdugo-Rodríguez A, Hernández-Chiñas U, Navarro-Ocaña A, Ahumada-Cota RE, et al. Pet dogs potential transmitters of pathogenic Escherichia coli with resistance to antimicrobials. Arch Microbiol. 2020 Jul;202(5):1173–9.

13.	Clermont O, Bonacorsi S, Bingen E. Rapid and Simple Determination of the *Escherichia coli* Phylogenetic Group. Appl Environ Microbiol. 2000 Oct;66(10):4555–8.

14.	Chaudhuri RR, Henderson IR. The evolution of the Escherichia coli phylogeny. Infect Genet Evol. 2012 Mar;12(2):214–26.

15.	Zhu Z, Jiang S, Qi M, Liu H, Zhang S, Liu H, et al. Prevalence and characterization of antibiotic resistance genes and integrons in Escherichia coli isolates from captive non-human primates of 13 zoos in China. Sci Total Environ. 2021 Dec;798:149268.

16.	Zhu Z, Pan S, Wei B, Liu H, Zhou Z, Huang X, et al. High prevalence of multi-drug resistances and diversity of mobile genetic elements in Escherichia coli isolates from captive giant pandas. Ecotoxicol Environ Saf. 2020 Jul;198:110681.

17.	Čurová K, Slebodníková R, Kmeťová M, Hrabovský V, Maruniak M, Liptáková E, et al. Virulence, phylogenetic background and antimicrobial resistance in Escherichia coli associated with extraintestinal infections. J Infect Public Health. 2020 Oct;13(10):1537–43.

18.	Kim B, Kim JH, Lee Y. Virulence Factors Associated With *Escherichia coli* Bacteremia and Urinary Tract Infection. Ann Lab Med. 2022 Mar 1;42(2):203–12.

19.	Zhang S, Chen S, Rehman MU, Yang H, Yang Z, Wang M, et al. Distribution and association of antimicrobial resistance and virulence traits in Escherichia coli isolates from healthy waterfowls in Hainan, China. Ecotoxicol Environ Saf. 2021 Sep;220:112317.

20.	Johnson JR, Russo TA. Molecular epidemiology of extraintestinal pathogenic (uropathogenic) Escherichia coli. Int J Med Microbiol. 2005 Oct;295(6–7):383–404.

21.	Gomes TAT, Elias WP, Scaletsky ICA, Guth BEC, Rodrigues JF, Piazza RMF, et al. Diarrheagenic Escherichia coli. Braz J Microbiol. 2016 Dec;47:3–30.

22.	Algammal AM, El-Tarabili RM, Alfifi KJ, Al-Otaibi AS, Hashem MEA, El-Maghraby MM, et al. Virulence determinant and antimicrobial resistance traits of Emerging MDR Shiga toxigenic E. coli in diarrheic dogs. AMB Express. 2022 Mar 17;12(1):34.

23.	Puño-Sarmiento J, Medeiros L, Chiconi C, Martins F, Pelayo J, Rocha S, et al. Detection of diarrheagenic Escherichia coli strains isolated from dogs and cats in Brazil. Vet Microbiol. 2013 Oct;166(3–4):676–80.

24.	Karczmarczyk M, Abbott Y, Walsh C, Leonard N, Fanning S. Characterization of Multidrug-Resistant Escherichia coli Isolates from Animals Presenting at a University Veterinary Hospital. Appl Environ Microbiol. 2011 Oct 15;77(20):7104–12.

25.	Boerlin P, Travis R, Gyles CL, Reid-Smith R, Heather Lim NJ, Nicholson V, et al. Antimicrobial Resistance and Virulence Genes of *Escherichia coli* Isolates from Swine in Ontario. Appl Environ Microbiol. 2005 Nov;71(11):6753–61.

26.	Yang GY, Guo L, Su JH, Zhu YH, Jiao LG, Wang JF. Frequency of Diarrheagenic Virulence Genes and Characteristics in Escherichia coli Isolates from Pigs with Diarrhea in China. Microorganisms. 2019 Sep 2;7(9):308.

27.	Guardabassi L. Pet animals as reservoirs of antimicrobial-resistant bacteria: Review. J Antimicrob Chemother. 2004 Jul 1;54(2):321–32.

28.	Wang B, Li S, Liang G, Li J, Li Y. Current status and prospects of pet medical development in Sichuan province. Anim. Agriculture. 2023 418 ;(01): 59-62.

29.	Fan S, Jiang S, Luo L, Zhou Z, Wang L, Huang X, et al. Antibiotic-Resistant Escherichia coli Strains Isolated from Captive Giant Pandas: A Reservoir of Antibiotic Resistance Genes and Virulence-Associated Genes. Vet Sci. 2022 Dec 18;9(12):705.

30.	So JH, Kim J, Bae IK, Jeong SH, Kim SH, Lim S kyung, et al. Dissemination of multidrug-resistant Escherichia coli in Korean veterinary hospitals. Diagn Microbiol Infect Dis. 2012 Jun;73(2):195–9.

31.	Gozi KS, Froes JR, Deus Ajude LPT, da Silva CR, Baptista RS, Peiró JR, et al. Dissemination of Multidrug-Resistant Commensal Escherichia coli in Feedlot Lambs in Southeastern Brazil. Front Microbiol. 2019 Jun 25;10:1394.

32.	Naziri Z, Poormaleknia M, Ghaedi Oliyaei A. Risk of sharing resistant bacteria and/or resistance elements between dogs and their owners. BMC Vet Res. 2022 Dec;18(1):203.

33.	Tuerena I, Williams NJ, Nuttall T, Pinchbeck G. Antimicrobial‐resistant *Escherichia coli* in hospitalised companion animals and their hospital environment. J Small Anim Pract. 2016 Jul;57(7):339–47.

34.	Saputra S, Jordan D, Mitchell T, Wong HS, Abraham RJ, Kidsley A, et al. Antimicrobial resistance in clinical Escherichia coli isolated from companion animals in Australia. Vet Microbiol. 2017 Nov;211:43–50.

35.	Scott Weese J. Antimicrobial resistance in companion animals. Anim Health Res Rev. 2008 Dec;9(2):169–76.

36.	Mustapha M, Audu Y, Ezema KU, Abdulkadir JU, Lawal JR, Balami AG, et al. Antimicrobial Susceptibility Profiles of *Escherichia Coli* Isolates from Diarrheic Dogs in Maiduguri, Borno State, Nigeria. Maced Vet Rev. 2021 Mar 1;44(1):47–53.

37.	Feng Y, Qin Z, Geng Y, Huang X, Ouyang P, Chen D, et al. Regional analysis of the characteristics and potential risks of bacterial pathogen resistance under high-pressure antibiotic application. J Environ Manage. 2022 Sep;317:115481.

38.	Fernandes V, Cunha E, Nunes T, Silva E, Tavares L, Mateus L, et al. Antimicrobial Resistance of Clinical and Commensal Escherichia coli Canine Isolates: Profile Characterization and Comparison of Antimicrobial Susceptibility Results According to Different Guidelines. Vet Sci. 2022 Jun 9;9(6):284.

39.	Zhang S, Chen S, Abbas M, Wang M, Jia R, Chen S, et al. High incidence of multi-drug resistance and heterogeneity of mobile genetic elements in Escherichia coli isolates from diseased ducks in Sichuan province of China. Ecotoxicol Environ Saf. 2021 Oct;222:112475.

40.	Van Den Bogaard A. Epidemiology of resistance to antibiotics Links between animals and humans. Int J Antimicrob Agents. 2000 May;14(4):327–35.

41.	Schmidt VM, Pinchbeck G, McIntyre KM, Nuttall T, McEwan N, Dawson S, et al. Routine antibiotic therapy in dogs increases the detection of antimicrobial-resistant faecal Escherichia coli. J Antimicrob Chemother [Internet]. 2018 Sep 11 [cited 2023 Apr 22]; Available from: https://academic.oup.com/jac/advance-article/doi/10.1093/jac/dky352/5095201
